# Studies on lipase-catalyzed asymmetric synthesis of (S)-(hydroxymethyl)glutamic acid (HMG)

**DOI:** 10.1186/s40064-015-1503-8

**Published:** 2015-11-24

**Authors:** Hiromasa Yoshioka, Masato Oikawa

**Affiliations:** Yokohama City University, Seto 22-2, Kanazawa-ku, Yokohama, 236-0027 Japan

**Keywords:** Glutamate analogs, Lipase-catalyzed acetylation, (Hydroxymethyl)glutamic acid, Asymmetric synthesis

## Abstract

(*S*)-(Hydroxymethyl)glutamic acid was successfully synthesized in total 12 % yield over eight steps from tris(hydroxymethyl)aminomethane hydrochloride (Tris·HCl), employing lipase TL-induced enantioselective acetylation of a prochiral 1,3-diol as the key step.

**Electronic supplementary material:**

The online version of this article (doi:10.1186/s40064-015-1503-8) contains supplementary material, which is available to authorized users.

## Background

**Fig. 1:**
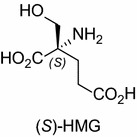
(*S*)-(Hydroxymethyl)glutamic acid ((*S*)-HMG), the group II mGluR ligand

The metabotropic glutamate receptors (mGluRs) play an important role in the modulation of synaptic transmission and neuronal excitability by glutamate, the main excitatory neurotransmitter, in the central nervous system (CNS) (Niswender and Conn 2010; Rondard and Pin 2015). mGluRs are members of the G-protein-coupled receptor (GPCR) superfamily, and belong to family C receptors that typically contain the endogenous ligand-binding site at a large extracellular *N*-terminal domain. mGluRs are subdivided into three groups, group I (mGluRs 1 and 5), group II (mGluRs 2 and 3), and Group III (mGluRs 4, 6, 7, and 8). Group II mGluRs reduce cAMP accumulation resulting in neuroprotecting effect and are closely linked to construction of memory and learning (Kawasaki et al. 2003). (Hydroxymethyl)glutamic acid (HMG) is one of the selective ligands for group II mGluRs. (*R*)-HMG is a selective agonist for mGluR3 (Miyaoka et al. 2006), and the (*S*)-counterpart [(*S*)-HMG, Fig. 1] has been shown to act as a more potent agonist for mGluR3 and a weak antagonist for mGluR2, both belong to group II (Choudhury et al. 2002).

Several practical synthetic studies of HMG have been so far reported. In 2001, the first synthesis of HMG has been reported by Kozikowski group (Zhang et al. 2001) employing Michael addition followed by ring closure. The same strategy has been employed in the synthesis reported by the group led by Jimenez-Oses and Avenoza (Aydillo et al. 2011). Synthesis by Strecker reaction (Choudhury et al. 2002; Kawasaki et al. 2003; Tang et al. 2004) has been proven to be also powerful. A number of other synthetic studies includes aldol reaction (Battistini et al. 2004), C–H insertion (Hayes et al. 2006), lipase-mediated acetylation of cyclic substrate (Miyaoka et al. 2006), chiral auxiliary-assisted diastereoselective alkylation (Yiotakis et al. 2007), and selective transformation of xylofuranose used as a chiral pool (Martinkova et al. 2008), as the key steps.

We have been also interested in the synthesis of the neuronally active compounds, by the divergent route amenable to the structural analogs to discover novel compounds (Chiba et al. 2015; Juknaitė et al. 2013; Oikawa et al. 2013; Sakai et al. 2014; Sugeno et al. 2014; Tanaka et al. 2015). Here, we report our synthetic study using lipase-mediated esterification of prochiral acyclic 1,3-diol as the key step. Although such enzymatic strategy has been previously reported as noted above (Miyaoka et al. 2006), in this study, we intended development of our own route not only to HMG but also to the analogs. Furthermore, enzymatic approach was expected to be applicable to the other biologically interesting natural products such as dysibetaine (Sakai et al. 1999) and sphingofungin E (Horn et al. 1992), by virtue of (1) the high catalytic activity and the enantioselectivity for a variety of substrates, and (2) the easy handling even in a large-scale synthesis.

## Methods

All reactions susceptible to moisture and air were carried out in an atmosphere of argon gas, using the glassware oven-dried over 3 h, and in the solvents freshly distilled from sodium and benzophenone. All other chemicals were purchased at the highest commercial grade and used directly. Lipase TL was kindly provided by Meito Sangyo Co., Ltd., Japan. Analytical thin-layer chromatography (TLC) was performed using Merck silica gel 60 F254 plate (0.25-mm thickness). Flash column chromatography was carried out using Merck silica gel 60 (230–400 mesh) or Fuji Silysia silica gel BW-300 (200–400 mesh). Reversed-phase silica gel column chromatography was carried out using Fuji Silysia Chromatorex DM1020T (0.10-mm thickness). For high-performance liquid chromatography (HPLC), recycling preparative system LC-918 (Japan Analytical Industries) was used. The analytical and preparative chiral HPLC experiments were performed on a JASCO PU-2080 system equipped with ELS-2041, using CHIRALPAK IC column (0.46 ×25 mm). IR spectra were recorded on a PerkinElmer Spectrum One FT-IR spectrometer. ^1^H and ^13^C NMR spectra were recorded on a BRUKER AVANCE 400 spectrometer. Chemical shift values are reported in δ (ppm) with reference to internal residual solvent [^1^H NMR, CDCl_3_ (7.24), D_2_O (4.70); ^13^C NMR, CDCl_3_ (77.0)]. Coupling constants (*J*) are reported in Hertz (Hz). The following abbreviations were used to designate the multiplicities; s = singlet, d = doublet, t = triplet, q = quartet, m = multiplet, br = broad.

### *tert-Butyl (5-(hydroxymethyl)-2-phenyl-1,3-dioxan-5-yl)carbamate (****2a****)*

To a stirred suspension of tris(hydroxymethyl)aminomethane hydrochloride (Tris·HCl, **1**, 30.1 g, 191 mmol) in DMF (200 mL) at rt were added TsOH·H_2_O (1.82 g, 9.54 mmol) and benzaldehyde dimethyl acetal (29.1 mL, 0.210 mmol). After being stirred for 24 h, to the resulting clear and colorless solution was added Et_3_N (1.6 mL, 11 mmol) and stirring was continued for additional 10 min. The mixture was then concentrated in vacuo, and Et_3_N (21.0 mL, 149 mmol) and EtOAc (750 mL) were added. White precipitate was removed by filtration, and the filtrate was concentrated in vacuo to afford crude benzylidene acetal (36.6 g) as a yellow oil.

To a stirred solution of the crude amine thus obtained above in water (87 mL) and 1,4-dioxane (87 mL) at rt was added K_2_CO_3_ (72.5 g, 525 mmol). After being stirred for 30 min, Boc_2_O (42.0 g, 192 mmol) was added. After 2 h, the mixture was poured into saturated aqueous NH_4_Cl (30 mL). The aqueous layer was separated and extracted with EtOAc (3 ×300 mL). The combined extracts were washed with brine (50 mL), dried over Na_2_SO_4_, and concentrated in vacuo. The residue was purified by precipitation from EtOAc (50 mL) to give diastereomerically pure *N*-Boc-protected benzylidene acetal **2a** (30.7 g, 52 % for two steps) as a white solid: IR (KBr) 3445, 3259, 2979, 1682 cm^−1^; ^1^H NMR (400 MHz, CDCl_3_) δ 7.51–7.33 (m, 5H), 5.45 (s, 1H), 4.19 (d, *J* = 11.6 Hz, 2H), 3.82 (d, *J* = 11.7 Hz, 2H), 3.69 (s, 2H), 1.45 (s, 9H); ^13^C NMR (100 MHz, CDCl_3_) δ 156.8, 137.5, 129.3 (×2), 128.4, 126.0 (×2), 102.0, 80.7, 71.8, 64.8, 53.6 (×2), 28.4 (×3). The spectroscopic data were identical to those reported (Ko et al. 2011; Schmidt and Riedl 1993).

### *tert*-*Butyl (5*-*formyl*-*2*-*phenyl*-*1,3*-*dioxan*-*5*-*yl)carbamate (****3****)*

To a stirred solution of alcohol **2a** (587 mg, 1.90 mmol) in CH_2_Cl_2_ (20 mL) at rt were added powdered MS4A (activated, 1.0 g), NMO (445 mg, 3.80 mmol), and TPAP (34.0 mg, 0.0954 mmol). After 6 h, insoluble materials were removed by filtration through a pad of Celite, and the filtrate was concentrated in vacuo. The residue was purified by silica gel column chromatography (300 g, EtOAc/hexane = 2:8) to give diastereomerically pure aldehyde **3** (350 mg, 60 %) as a white solid: IR (KBr) 3440, 3414, 2977, 1726, 1692 cm^−1^; ^1^H NMR (400 MHz, CDCl_3_) δ 9.51 (s, 1H), 7.49–7.36 (m, 5H), 5.44 (s, 1H), 4.23 (d, *J* = 11.4 Hz, 2H), 4.07 (d, *J* = 11.2 Hz, 2H), 1.46 (s, 9H); ^13^C NMR (100 MHz, CDCl_3_) δ 198.7, 155.8, 137.2, 129.4 (×2), 128.4 (×2), 126.0, 101.5, 80.9, 69.4, 60.4 (×2), 28.3 (×3). The spectroscopic data were identical to those reported (Ko et al. 2011; Schmidt and Riedl 1993).

### *(E)*-*tert*-*Butyl 3*-*(5*-*((tert*-*butoxycarbonyl)amino)*-*2*-*phenyl*-*1,3*-*dioxan*-*5*-*yl)acrylate (****4****)*

To a stirred solution of aldehyde **3** (350 mg, 1.14 mmol) in CH_2_Cl_2_ (10 mL) at 35 °C were added *tert*-butyl (triphenylphosphoranylidene)acetate (643 mg, 1.71 mmol). After 2 h, the mixture was concentrated in vacuo. The residue was purified by silica gel column chromatography (10 g, EtOAc/hexane = 2:8) to give diastereomerically pure α,β-unsaturated ester **4** (422 mg, 92 %) as a white solid: IR (KBr) 3385, 2979, 2932, 2870, 1706 cm^−1^; ^1^H NMR (400 MHz, CDCl_3_) δ 7.54–7.40 (m, 5H), 6.80 (d, *J* = 16.2 Hz, 1H), 5.94 (d, *J* = 16.2 Hz, 1H), 5.52 (s, 1H), 4.33 (d, *J* = 10.6 Hz, 2H), 3.89 (d, *J* = 11.5 Hz, 2H), 1.51 (s, 9H), 1.48 (s, 9H); ^13^C NMR (100 MHz, CDCl_3_) δ 165.1, 154.7, 143.6, 137.3, 129.4, 128.4 (×2), 126.0 (×2), 124.2, 101.8, 80.9, 80.1, 72.1, 53.2 (×2), 28.4 (×3), 28.1 (×3).

### *tert*-*Butyl 4*-*((tert*-*butoxycarbonyl)amino)*-*5*-*hydroxy*-*4*-*(hydroxymethyl)pentanoate (****5****)*

To a solution of alkenyl acetal **4** (422 mg, 1.04 mmol) in MeOH (10 mL) at rt was added Pd/C (10 % w/w, 40 mg), and the flask was purged with H_2_. After stirring for 3 h, the mixture was filtered through a pad of Celite using MeOH (10 mL). The solvent was removed under reduced pressure, and the residue was purified by silica gel column chromatography (3 g, EtOAc/hexane = 1:1) to give diol **5** (194 mg, 58 %) as a white solid: IR (film) 3358, 2978, 2363, 1714 cm^−1^; ^1^H NMR (400 MHz, CDCl_3_) δ 3.61 (d, *J* = 11.9 Hz, 2H), 3.47 (d, *J* = 11.9 Hz, 2H), 2.30 (t, *J* = 6.8 Hz, 2H), 1.93 (t, *J* = 6.8 Hz, 2H), 1.42 (s, 9H), 1.40 (s, 9H); ^13^C NMR (100 MHz, CDCl_3_) δ 174.4, 156.4, 81.4, 80.3, 65.3 (×2), 29.5, 28.3 (×3), 28.3 (×3), 28.0, 26.3.

### *tert*-*Butyl (R)*-*5*-*acetoxy*-*4*-*((tert*-*butoxycarbonyl)amino)*-*4*-*(hydroxymethyl)pentanoate (****6****)* (lipase TL-catalyzed enzymatic reaction, entry 8 in Table 1)

To a stirred solution of diol **5** (4.59 mg, 0.0144 mmol) in CH_2_Cl_2_ (0.600 mL) at rt were added lipase TL (5.03 mg) and vinyl acetate (0.00265 mL, 0.0287 mmol). After 3 days, insoluble materials were removed by filtration through a pad of Celite, and the filtrate was concentrated in vacuo. Chiral HPLC analysis [Chiralcel IC column, 20 % ethanol in hexane, 1.0 mL/min, λ = 210 nm, tR = 7.0, 7.5 (major)] of the residue showed that the reaction proceeded in 33 % yield with 88:12 enantioselectivity. The crude material was purified by silica gel column chromatography (500 mg, EtOAc/hexane = 4:6) to give monoacetate **6** (1.60 mg, 0.00443 mmol, 30 %) as a colorless oil, which was further purified to be 100 % ee by preparative chiral HPLC.

**Table 1:** Screening of lipases for enantioselective acetylation of diol **5** ^a^

Entry	Lipase	Time (h)	Conversion (%)^b^	Ee of 6 (%)^c^
1	AK	21	N.R.	–
2	PS-IM	48	N.R.	–
3	XP-488	72	N.R.	–
4	OF	24	N.R.	–
5	QLM	72	59	47
6	PL	24	29	41
7	Novozyme 435	24	42	9.5
8	TL	72	33	76

*N.R.* no reaction

^a^Two equiv of vinyl acetate, and a same amount of lipase, as diol **5**, were used

^b^Determined by ^1^H NMR

^c^Determined by chiral HPLC analysis

Data for monoacetate **6** (100 % ee): [α]_D_^25^ −18.1 (*c* 0.064, CHCl_3_); IR (film) 3361, 2978, 2931, 2360, 1722 cm^−1^; ^1^H NMR (400 MHz, CDCl_3_) δ 4.28 (d, *J* = 11.3 Hz, 1H), 4.14 (d, *J* = 11.3 Hz, 1H), 3.63 (d, *J* = 11.8 Hz, 1H), 3.55 (d, *J* = 11.8 Hz, 1H), 2.36–2.22 (m, 2H), 2.08 (s, 3H), 2.05 (m, 1H), 1.85 (m, 1H), 1.43 (s, 9H), 1.41 (s, 9H); ^13^C NMR (100 MHz, CDCl_3_) δ 173.1, 171.1, 155.7, 81.0, 80.3, 77.3, 65.0, 58.1, 29.3, 28.3 (×3), 28.1 (×3), 27.6, 20.9.

### *5*-*(tert*-*Butoxy)*-*2*-*((tert*-*butoxycarbonyl)amino)*-*2*-*(hydroxymethyl)*-*5*-*oxopentyl hexanoate (****7****)* (lipase TL-catalyzed enzymatic reaction, entry 3 in Table 2)

To a stirred solution of diol **5** (9.6 mg, 0.030 mmol) in CH_2_Cl_2_ (0.600 mL) at rt were added lipase TL (9.8 mg) and vinyl hexanoate (0.0096 mL, 0.063 mmol). After 2 days, the reaction mixture was warmed to 35 °C. After 6 h, insoluble materials were removed by filtration through a pad of Celite, and the filtrate was concentrated in vacuo. Chiral HPLC analysis [Chiralcel IC column, 20 % ethanol in hexane, 1.0 mL/min, λ = 210 nm, tR = 5.8, 6.9 (major)] of the residue showed that the reaction proceeded in 34 % yield with 84.9:15.1 enantioselectivity. The crude material was purified by silica gel column chromatography (500 mg, EtOAc/hexane = 3:7) to give monohexanoate **7** (70 % ee, 4.3 mg, 0.010 mmol, 34 %) as a white solid: [α]_D_^22^ −3.45 (*c* 0.21, CHCl_3_); IR (film) 3367, 2931, 2360, 1722, 1509, 1367 cm^−1^; ^1^H NMR (400 MHz, CDCl_3_) δ 4.88 (br, 1H), 4.27 (d, *J* = 11.3 Hz, 1H), 4.12 (d, *J* = 11.3 Hz, 1H), 3.61 (d, *J* = 12.0 Hz, 1H), 3.54 (d, *J* = 12.2 Hz, 1H), 2.32 (t, *J* = 7.6 Hz, 2H), 2.26 (t, *J* = 7.3 Hz, 2H), 2.06 (m, 1H), 1.85 (m, 1H), 1.61 (t, *J* = 7.4 Hz, 2H), 1.42 (s, 9H), 1.41 (s, 9H), 1.33–1.24 (m, 4H), 0.88 (t, *J* = 6.9 Hz, 3H); ^13^C NMR (100 MHz, CDCl_3_) δ 173.9, 173.1, 155.7, 81.0, 77.2, 65.0, 64.8, 58.2, 34.2, 31.3, 29.4, 28.3 (×3), 28.1 (×3), 27.6, 24.6, 22.3, 13.9.

**Table 2:** Screening of solvents in acetylation of diol **5** mediated by lipase TL^a^

Entry	Solvent (log *P*)	Time (h)	Isolated yield (%)	Ee of 6 (%)^f^
1^b^	CH_2_Cl_2_ (1.25^c^)	72	30	76
2	Hexane (3.5^c^)/THF (0.49^d^) (1:1)	14	32	63
3	iPr_2_O (1.9^d^)	48	50	62
4	Benzene (2.0^c^)	15	60	38
5	Vinyl acetate (0.629 ± 0.286^e^)	2.5	63	64

^a^Two equiv of vinyl acetate (except for entry 5), and a same amount of lipase, as diol **5**, were used

^b^Same reaction shown in Table 1, entry 8

^c^Taken from the recent paper by Salihu and Alam (2015)

^d^Taken from Lee’s paper (Lee et al. 2004)

^e^A calculated value taken from SciFinder Scholar (April 17, 2015)

^f^Determined by chiral HPLC analysis

### *(S)*-*2*-*(Acetoxymethyl)*-*5*-*(tert*-*butoxy)*-*2*-*((tert*-*butoxycarbonyl)amino)*-*5*-*oxopentanoic acid (****8****)*

To a vigorously stirred solution of alcohol **6** (6.56 mg, 0.0157 mmol) in acetone (0.200 mL) at rt was added a suspension of sodium metaperiodate (42.7 mg, 0.199 mmol) and RuCl_3_·nH_2_O (1.30 mg, 0.00628 mmol) in water (0.200 mL). After 2 h, acetone (2 mL) was added and organic layer was separated. Insoluble Ru species were removed by filtration through a pad of Celite. The filtrate was concentrated in vacuo to give crude carboxylic acid **8** (7.61 mg) as a clear oil: ^1^H NMR (400 MHz, CDCl_3_) δ 4.70 (d, *J* = 12.1 Hz, 2H), 4.42 (br, 1H), 2.43–2.06 (m, 4H), 2.04 (s, 3H), 1.42 (br s, 18H).

### *(S)*-*2*-*Amino*-*2*-*(hydroxymethyl)pentanedioic acid hydrochloride ((S)*-*2*-*(hydroxymethyl)glutamic acid hydrochloride, (S)*-*HMG·HCl,***9***)*

A suspension of crude *N*-Boc-amino ester **8** (7.61 mg) thus obtained above in hydrochloric acid (6 M, 2.00 mL) was stirred at reflux for 25 h. The reaction mixture was then concentrated in vacuo. The residue was purified by column chromatography on reversed-phase silica gel (500 mg, water) to give (*S*)-2-amino-2-(hydroxymethyl)pentanedioic acid hydrochloride ((*S*)-HMG·HCl, **9**, 3.02 mg, 78 % for two steps from **6**) as a white solid: [α]_D_^24^ + 7.1 (*c* 0.15, H_2_O); ^1^H NMR (400 MHz, D_2_O) δ 3.81 (d, *J* = 12.0 Hz, 1H), 3.58 (d, *J* = 12.0 Hz, 1H), 2.42–2.23 (m, 2H), 2.00–1.83 (m, 2H); ^13^C NMR (100 MHz, D_2_O) δ 176.8, 173.2, 65.2, 63.8, 28.6, 27.1. The spectroscopic data were in good accord with those reported previously (Aydillo et al. 2011).

## Results and discussion

In the present study, we decided to construct the chiral center of HMG using lipase-catalyzed asymmetric reaction. The prochiral 1,3-diol, prepared from Tris·HCl, was designed as the substrate, and several lipases were planned to be screened. Further functional group transformation was expected to give enantiomerically pure HMG.

**Scheme 1:**
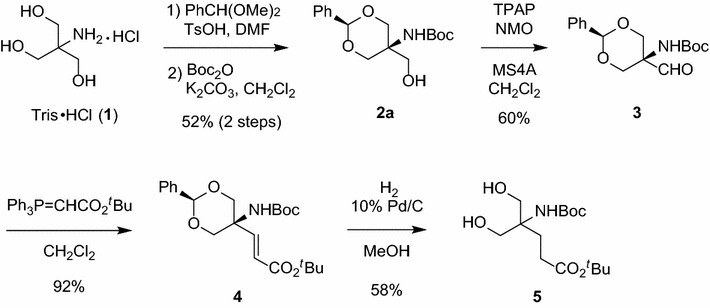
Synthesis of diol **5** as a substrate for enzymatic reaction

**Fig. 2:**
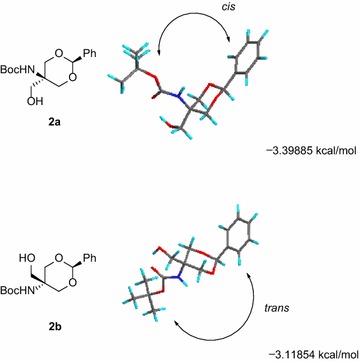
Lowest energy conformation of two possible diastereomers for benzylidene acetals **2a** and **2b**

As shown in Scheme 1, tris(hydroxymethyl)aminomethane hydrochloride (Tris·HCl, **1**) was converted to *N*-Boc-protected benzylidene acetal **2a** in 52 % yield over two steps including acetalization (PhCH(OMe)_2_, TsOH, DMF) and carbamate formation (Boc_2_O, K_2_CO_3_). Acetal **2a** was obtained as a single isomer, wherein the amino and phenyl groups are supposed to be in *cis* relationships from molecular modeling using CONFLEX (Fig. 2). Here, conformational searches were carried out with the MMFF94S force field to generate the lowest energy conformation for each isomer (Fig. 2a, b), and the steric energy indicated that **2a** is more stable by 0.28031 kcal/mol. Since benzylidene acetal formation was apparently a thermodynamically controlled process, more stable diastereomer **2a** was concluded to be likely. Unfortunately, no experimental or spectroscopic support is available so far; **2a** was not obtained as a crystal suitable for X-ray analysis, and no clear NOESY cross peak was observed for stereochemical assignment. Interestingly, acetal **2a**, as well as the next aldehyde **3** (see below), has been previously synthesized (Ko et al. 2011; Schmidt and Riedl 1993) by a different route without determination of the stereochemistry, and the reported spectroscopic data are identical to those for **2a** (and **3**) collected in the present study.

Alcohol **2a** was next converted to aldehyde **3** by Ley-Griffith oxidation (TPAP, NMO, MS4A) (Griffith et al. 1987; Ley et al. 1994) in 60 % yield. Wittig reaction of aldehyde **3** with *tert*-butyl (triphenylphosphoranylidene)acetate in CH_2_Cl_2_ gave α,β-unsaturated ester **4** in 92 % yield with exclusive (*E*)-selectivity. The molecular framework of HMG was thus constructed in these four-step reactions.

The next step is deprotection of benzylidene acetal and reduction of olefin. These two transformations were simultaneously performed under conditions for hydrogenation (H_2_, 10 % Pd/C, MeOH) to furnish prochiral 1,3-diol **5** in 68 % yield, ready for lipase-catalyzed desymmetrization.

Enantioselective desymmetrization of 1,3-diol **5** was explored using eight lipases as shown in Table 1. The lipases are stable in organic solvents and readily available, and thus were expected to allow the reaction to be performed in a large scale. All reactions were carried out using **5** (3.5–14 mg), lipase (same amount as **5**), and vinyl acetate (two equiv) at rt in solvents indicated. The reactions were conducted until the maximum conversion was achieved as judged from TLC analysis. It was found that lipases AK (Alexandre and Huet 1998), PS-IM (Hamada et al. 2010), XP-488 (Fujima et al. 2003), and OF (Chênevert et al. 2004) do not catalyze the reaction and 1,3-diol **5** was recovered intact (entries 1–4). When lipase QLM (Naemura et al. 1996) was used for 3 days, ^1^H NMR spectrum indicated the reaction proceeded at 59 % conversion (**5**/**6** = 41:59, entry 5). No other product such as diacetate was observed, and enantiomeric purity of monoacetate **6** was 47 % ee as determined by chiral HPLC analysis. The stereochemistry of **6** was not determined here, but was clarified later to be (*R*) configuration by leading to (*S*)-HMG (see below). In the following entries 6–8, the same enantioselectivity was observed. However, with lipases PL (Alexandre and Huet 1998) and Novozyme 435 (Chênevert et al. 2004), the enantioselectivity was decreased to be 41 % ee and 9.5 % ee, respectively (entries 6, 7). Fortunately, it was found that lipase TL (van Pelt et al. 2011) provides enantiomerically more pure monoacetate **6** (76 % ee) at 33 % conversion (30 % isolation yield) after 3 days (entry 8). Again, no diacetate was observed here chromatographically and spectroscopically, and unreacted 1,3-diol **5** was quantitatively recovered in all entries. We further studied enzymatic acylation using lipase TL to optimize reaction conditions.

Table 2 shows the isolation yield and the ee, in acetylation of **5** mediated by lipase TL, with a variety of solvents. The reaction in CH_2_Cl_2_, which has been shown in Table 1, entry 8, gave monoacetate **6** in 30 % isolated yield (76 % ee, entry 1). Mixed solvent of hexane and THF (1:1) was found to give **6** in comparable yield with decreased enantioselectivity (63 % ee, entry 2). Employment of iPr_2_O improved the yield to 50 %, while the level of ee was preserved (62 % ee, entry 3). Benzene as a solvent further improved the yield (60 %), but the enantioselectivity was largely diminished to 38 % ee (entry 4). In entry 5, vinyl acetate was employed as a solvent, wherein the highest yield (63 %) was observed with 64 % ee. In all entries no diacetate was obtained and unreacted diol **5** was recovered quantitatively. It is generally accepted that organic solvents with high log *P* values (octanol–water partition coefficient) are preferably used in lipase-catalyzed reaction, because of their poor ability to remove essential water molecules from lipase (Salihu and Alam 2015). In our case shown in Table 3, however, no obvious correlation was observed between the isolated yield and log *P* of the solvent.

**Table 3:** Screening of solvents for hexanoylation of diol **5** mediated by lipase TL^a^

Entry	Conditions	Conversion (%)^b^	Ee of 7 (%)^c^
1	iPr_2_O, rt → 50 °C, 19 h	30	6.4
2	Benzene, rt, 48 h	70	65
3	CH_2_Cl_2_, rt → 35 °C, 54 h	34	70

^a^Two equiv of vinyl hexanoate, and a same amount of lipase, as diol **5**, were used

^b^Determined by ^1^H NMR

^c^Determined by chiral HPLC analysis

Lipase TL also mediated esterification with longer acyl group (Tsuji et al. 2005) at, in some cases, elevated temperature (Table 3). Three solvents were examined with two equiv of vinyl hexanoate. Entry 1 shows hexanoylation in iPr_2_O. The reaction slowly proceeded at 50 °C over 19 h to give hexanoate **7** in 30 % conversion yield. Enantioselectivity was disappointingly low (6.4 % ee) as determined from chiral HPLC analysis. However, the results were improved when benzene was employed (entry 2); benzene allows hexanoylation at rt to provide **7** in 70 % yield with 65 % ee. Even higher enantioselectivity (70 % ee) was observed in CH_2_Cl_2_ (entry 3), whereas the yield decreased to 34 %. As for the enantioselectivity, thus, CH_2_Cl_2_ was found to be practical in both acetylation and hexanoylation.

In nature, lipases catalyze hydrolysis of ester to give carboxylic acid by (A) nucleophilic attack of the active site serine to the ester carbonyl group to form acyl-enzyme intermediate, (B) which then suffers hydrolysis (Ghanem 2007; Adlercreutz 2013). Transesterification (alcoholysis of ester) by lipase is believed to proceed by the same mechanism, and interactions between the acylated enzyme active site and the alcohol, involved in the second event (e.g. B), influence the rate of the reaction. The only modest enantioselectivities and yields observed in Tables 1, 2 and 3 would be due to rather severe interactions caused by sterically demanding alcohol substrate **5**.

**Scheme 2:**
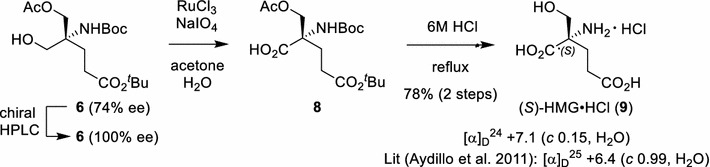
Final elaboration toward (*S*)-HMG (**9**)

With monoacetate **6** in hand, we continued the synthetic study toward 2-(hydroxymethyl)glutamic acid (HMG) (Scheme 2). The enantiomeric purity of **6** was first enhanced to 100 % ee by preparative chiral HPLC. The stereochemistry of **6** was expected to be clarified by comparison of the physical or chromatographic properties of our synthetic HMG with authentic specimens. Thus, RuO_4_-mediated oxidation (Oba et al. 2006) of alcohol **6** afforded carboxylic acid **8**, which is the protected precursor for HMG. Finally, *N*-Boc-amino ester **8** was hydrolyzed in refluxing 6 M hydrochloric acid to give HMG·HCl (**9**) in 78 % yield (two steps). Optical rotation data [(α)_D_^24^ + 7.1 (*c* 0.15, H_2_O)] indicated the (*S*) configuration (Aydillo et al. 2011). (*S*)-2-(Hydroxymethyl)glutamic acid hydrochloride (**9**) was thus synthesized in 12 % overall yield for eight steps starting from Tris·HCl.

## Conclusions

In this paper, we demonstrated enantioselective synthesis of (*S*)-HMG (**9**) employing lipase-catalyzed asymmetric esterification of prochiral 1,3-diol **5**. Overall yield was 12 % for total eight steps. As compared to the other shorter step syntheses of HMG (Zhang et al. 2001; Aydillo et al. 2011), our work is obviously not satisfactory (see Additional file 1 for summary for previous synthetic study of HMG). Nevertheless, we believe our present results are advantageous to provide the additional way to produce not only (*S*)-HMG but also the (*R*)-congener, since hydroxy groups of the intermediate **6** are orthogonally derivatized. Moreover, the present synthesis pathway is also applicable to other biologically important class of α,α-disubstituted amino acid such as dysibetaine (Sakai et al. 1999), sphingofungin E (Horn et al. 1992), and other related natural products. Works are in progress toward these compounds, and the results will be reported in due course.

## Additional file

10.1186/s40064-015-1503-8 NMR spectra for all new compounds, chiral HPLC profile for monoacetate **6**, and a summary for previous synthetic study of HMG.

## Abbreviations

CNS: central nervous system
GPCR: G-protein-coupled receptor
HMG: (hydroxymethyl)glutamic acid
HPLC: high-performance liquid chromatography
log P: octanol–water partition coefficient
mGluR: metabotropic glutamate receptor
TLC: thin-layer chromatography
Tris·HCl: tris(hydroxymethyl)aminomethane hydrochloride
