# Supplementary material for: Studies on lipase-catalyzed asymmetric synthesis of (S)-(hydroxymethyl)glutamic acid (HMG)
Source: Springerplus. 2015 Nov 24;4:726. doi: 10.1186/s40064-015-1503-8 (PMC4656251; doi:10.1186/s40064-015-1503-8)
Supplement: Supplementary file 1 — 10.1186/s40064-015-1503-8 NMR spectra for all new compounds, chiral HPLC profile for monoacetate 6, and a summary for previous synthetic study of HMG. [file 40064_2015_1503_MOESM1_ESM.pdf]

Supporting Information

Studies on lipase-catalyzed asymmetric synthesis of (*S*)-(hydroxymethyl)glutamic acid (HMG)

Hiromasa Yoshioka and Masato Oikawa*

Yokohama City University, Seto 22-2, Kanazawa-ku, Yokohama 236-0027, Japan

E-mail: moikawa@yokohama-cu.ac.jp

Contents:

NMR spectra for all new compounds **S3–S10**
Chiral HPLC profile for monoacetate **6** **S11**
A summary for previous synthetic study of HMG **S12–S13**

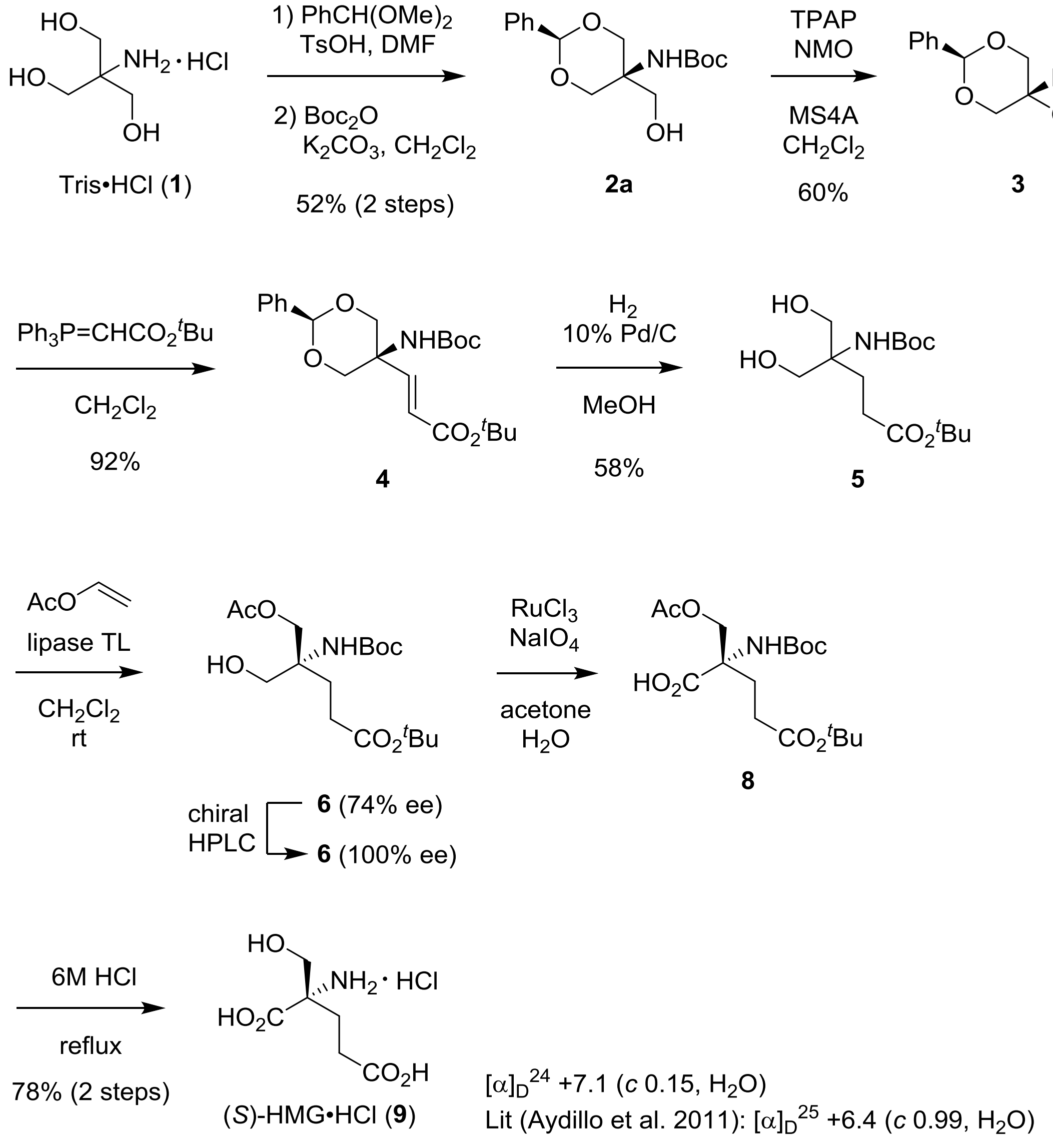

Scheme 1

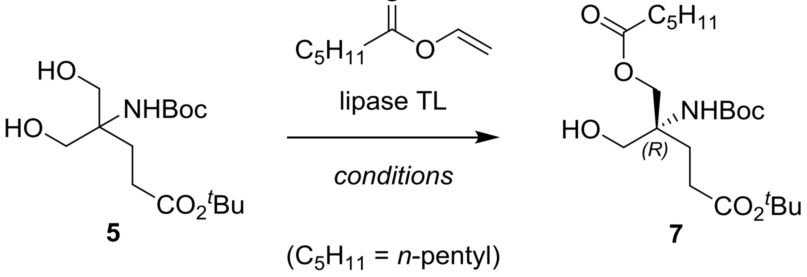

Scheme 2

1H NMR (400 MHz, CDCl3)

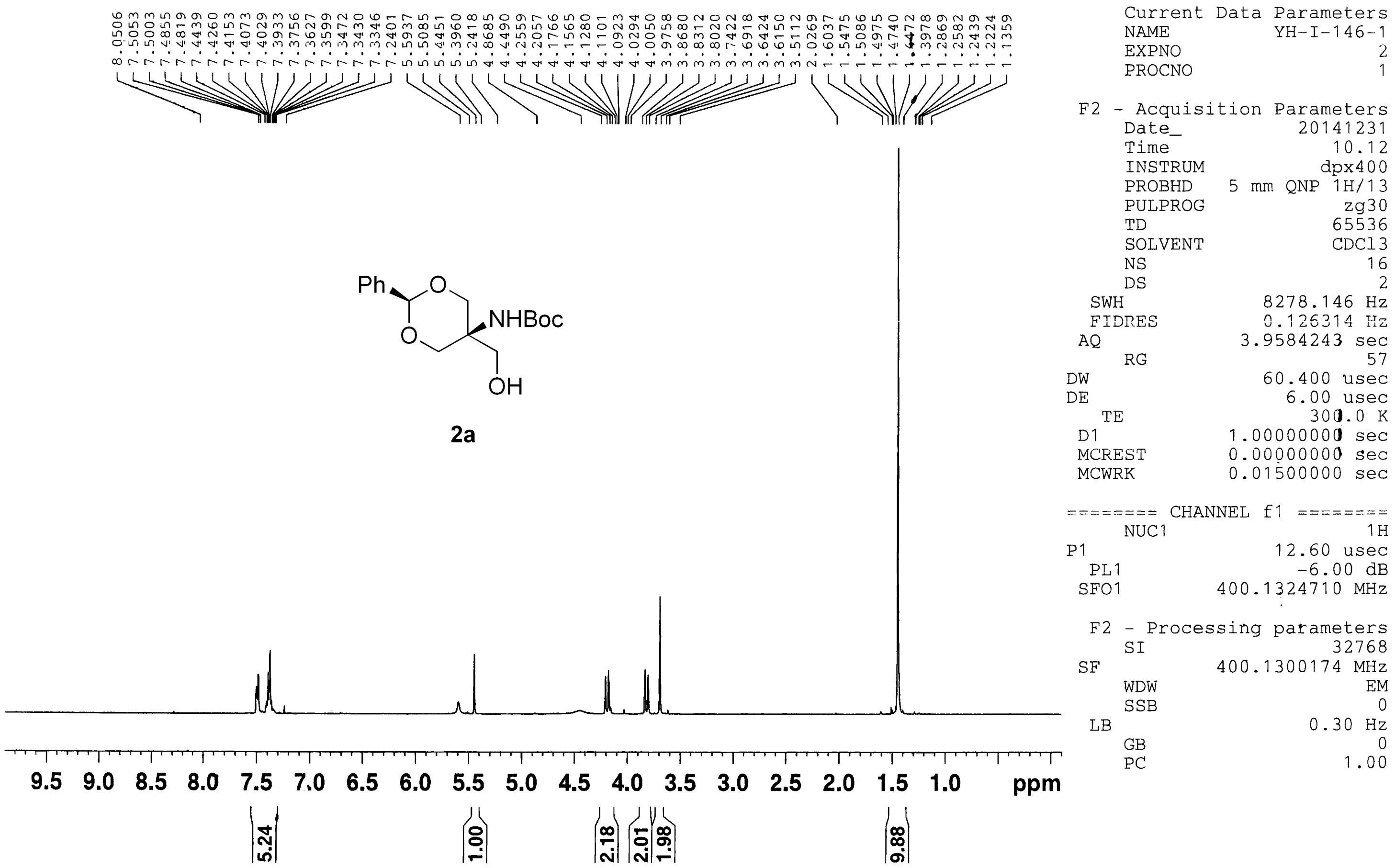

2a

13C NMR (100 MHz, CDCl3)

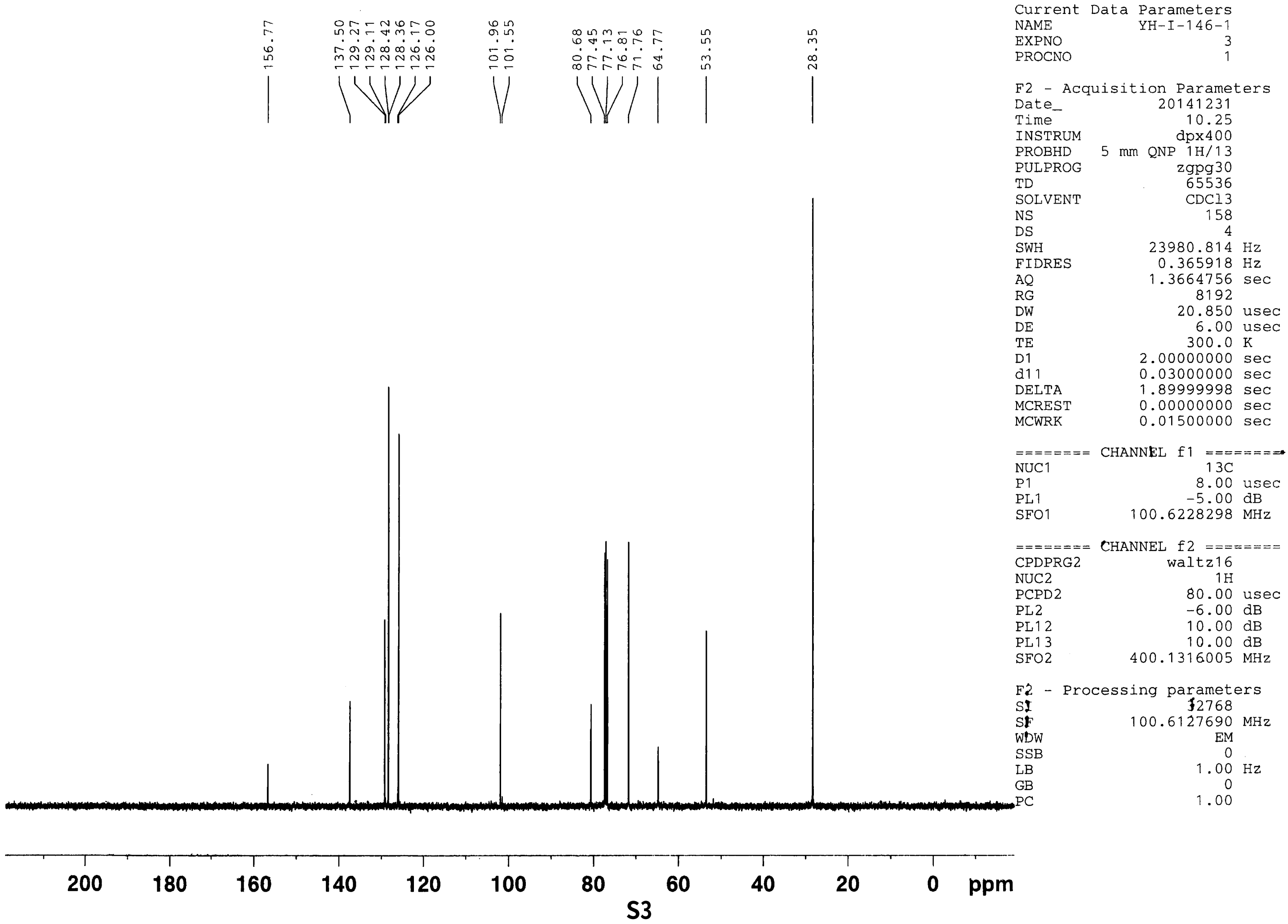

1H NMR (400 MHz, CDCl3)

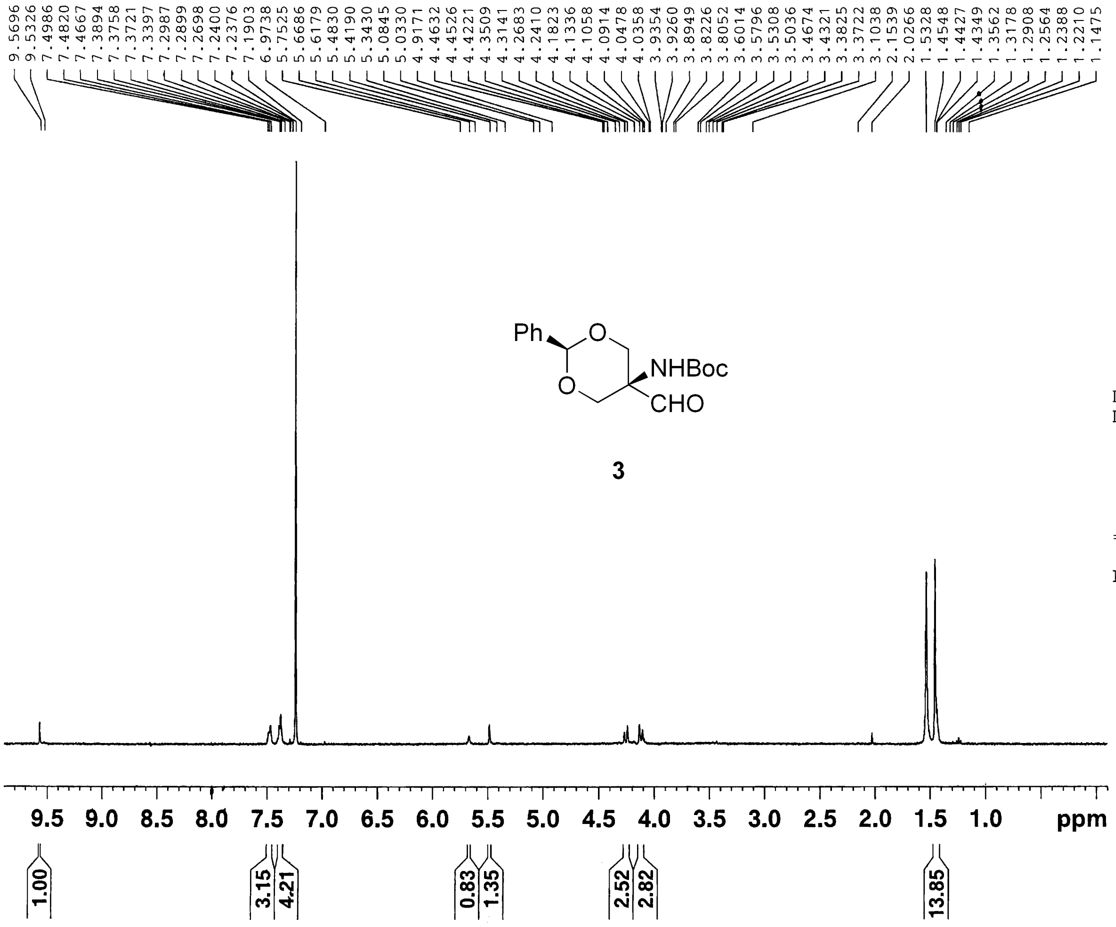

3

Current Data Parameters	
NAME	YH-I-147-1
EXPNO	1
PROCNO	1
F2 - Acquisition Parameters	
Date_	20141121
Time	12.57
INSTRUM	dpx400
PROBHD	5 mm QNP 1H/13
PULPROG	zg30
TD	65536
SOLVENT	CDCl3
NS	16
DS	2
SWH	8278.146 Hz
FIDRES	0.126314 Hz
AQ	3.9584243 sec
RG	1149.4
DW	60.400 usec
DE	6.00 usec
TE	300.0 K
D1	1.00000000 sec
MCREST	0.00000000 sec
MCWRK	0.01500000 sec
======== CHANNEL f1 ========	
NUC1	1H
P1	12.60 usec
PL1	-6.00 dB
SFO1	400.1324710 MHz
F2 - Processing parameters	
SI	32768
SF	400.1300179 MHz
WDW	EM
SSB	0
LB	0.30 Hz
GB	0
PC	1.00

13C NMR (100 MHz, CDCl3)

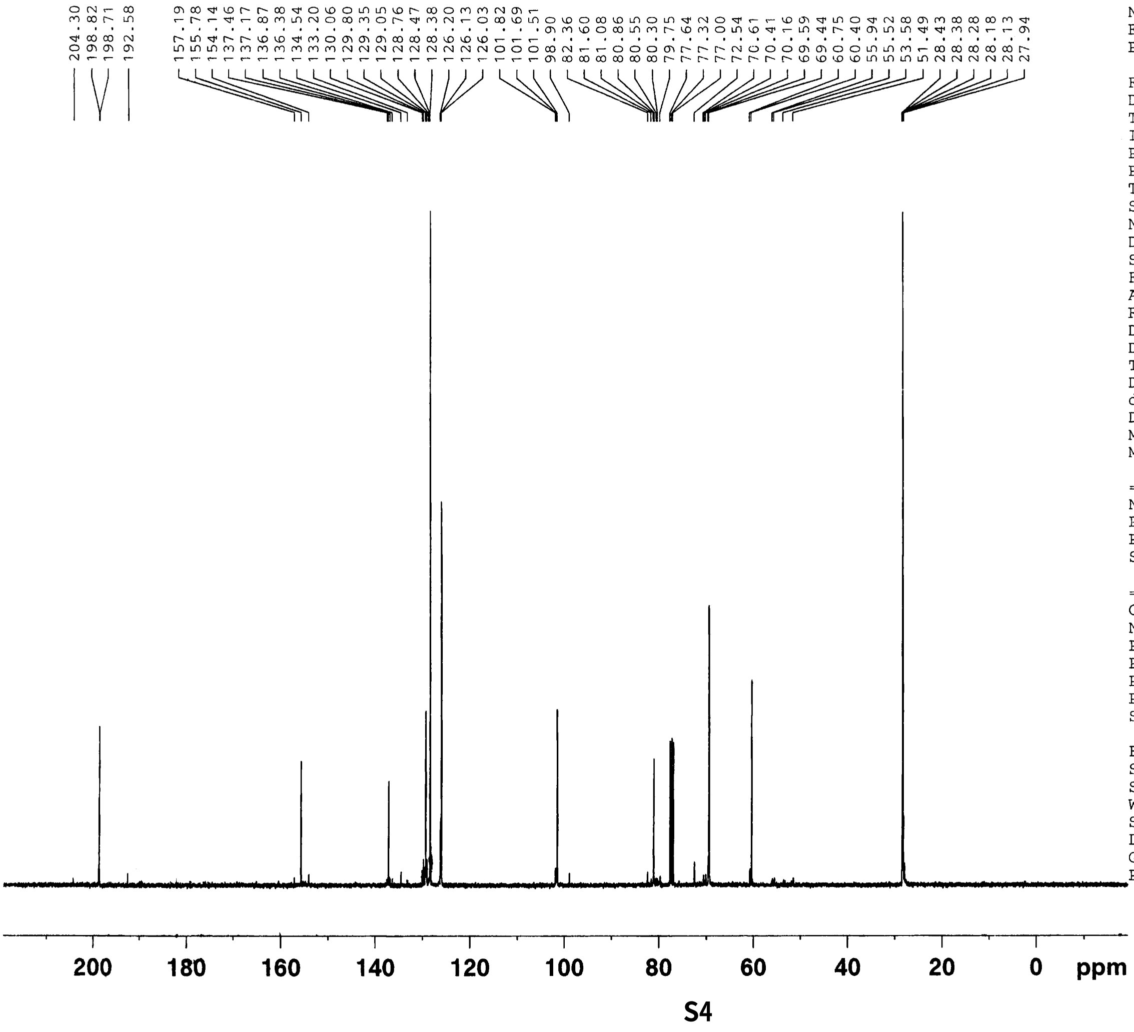

Current Data Parameters	
NAME	YH-I-147-1
EXPNO	3
PROCNO	1
F2 - Acquisition Parameters	
Date_	20141231
Time	10.49
INSTRUM	dpx400
PROBHD	5 mm QNP 1H/13
PULPROG	zgpg30
TD	65536
SOLVENT	CDCl3
NS	200
DS	4
SWH	23980.814 Hz
FIDRES	0.365918 Hz
AQ	1.3664756 sec
RG	6502
DW	20.850 usec
DE	6.00 usec
TE	300.0 K
D1	2.00000000 sec
d11	0.03000000 sec
DELTA	1.89999998 sec
MCREST	0.00000000 sec
MCWRK	0.01500000 sec
======== CHANNEL f1 ========	
NUC1	13C
P1	8.00 usec
PL1	-5.00 dB
SFO1	100.6228298 MHz
======== CHANNEL f2 ========	
CPDPRG2	waltz16
NUC2	1H
PCPD2	80.00 usec
PL2	-6.00 dB
PL12	10.00 dB
PL13	10.00 dB
SFO2	400.1316005 MHz
F2 - Processing parameters	
SI	32768
SF	100.6127690 MHz
WDW	EM
SSB	0
LB	1.00 Hz
GB	0
PC	1.00

1H NMR (400 MHz, CDCl3)

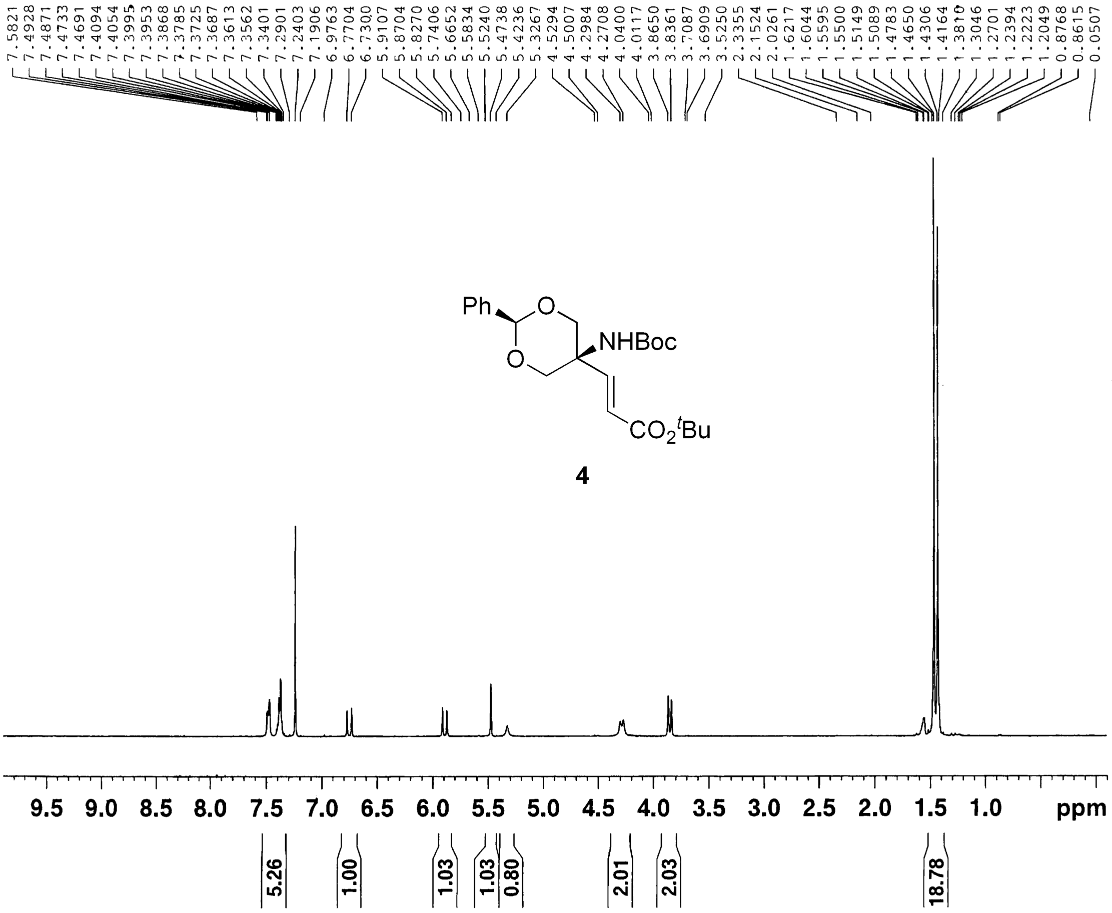

Current Data Parameters
NAME YH-I-165-1
EXPNO 1
PROCNO 1

F2 - Acquisition Parameters
Date_ 20141204
Time 18.38
INSTRUM dpx400
PROBHD 5 mm QNP 1H/13
PULPROG zg30
TD 65536
SOLVENT CDCl3
NS 16
DS 2
SWH 8278.146 Hz
FIDRES 0.126314 Hz
AQ 3.9584243 sec
RG 406.4
DW 60.400 usec
DE 6.00 usec
TE 300.0 K
D1 1.00000000 sec
MCREST 0.00000000 sec
MCWRK 0.01500000 sec

======== CHANNEL f1 ========
NUC1 1H
P1 12.60 usec
PL1 -6.00 dB
SFO1 400.1324710 MHz

F2 - Processing parameters
SI 32768
SF 400.1300174 MHz
WDW EM
SSB 0
LB 0.30 Hz
GB 0
PC 1.00

13C NMR (100 MHz, CDCl3)

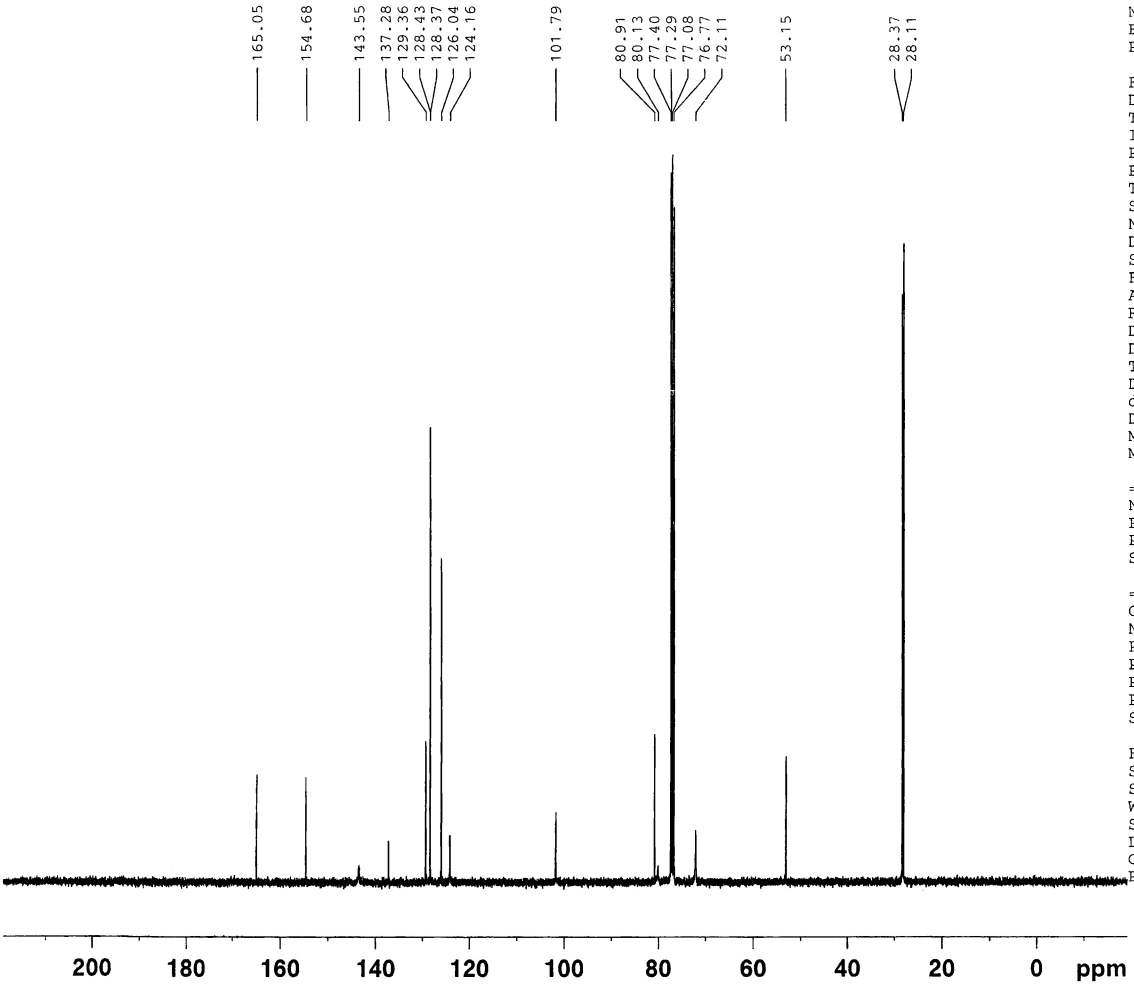

Current Data Parameters
NAME YH-I-165-1
EXPNO 3
PROCNO 1

F2 - Acquisition Parameters
Date_ 20150102
Time 12.13
INSTRUM dpx400
PROBHD 5 mm QNP 1H/13
PULPROG zgpg30
TD 65536
SOLVENT CDCl3
NS 835
DS 4
SWH 23980.814 Hz
FIDRES 0.365918 Hz
AQ 1.3664756 sec
RG 4597.6
DW 20.850 usec
DE 6.00 usec
TE 300.0 K
D1 2.00000000 sec
d11 0.03000000 sec
DELTA 1.89999998 sec
MCREST 0.00000000 sec
MCWRK 0.01500000 sec

======== CHANNEL f1 ========
NUC1 13C
P1 8.00 usec
PL1 -5.00 dB
SFO1 100.6228298 MHz

======== CHANNEL f2 ========
CPDPRG2 waltz16
NUC2 1H
PCPD2 80.00 usec
PL2 -6.00 dB
PL12 10.00 dB
PL13 10.00 dB
SFO2 400.1316005 MHz

F2 - Processing parameters
SI 32768
SF 100.6127690 MHz
WDW EM
SSB 0
LB 1.00 Hz
GB 0
PC 1.00

1H NMR (400 MHz, CDCl3)

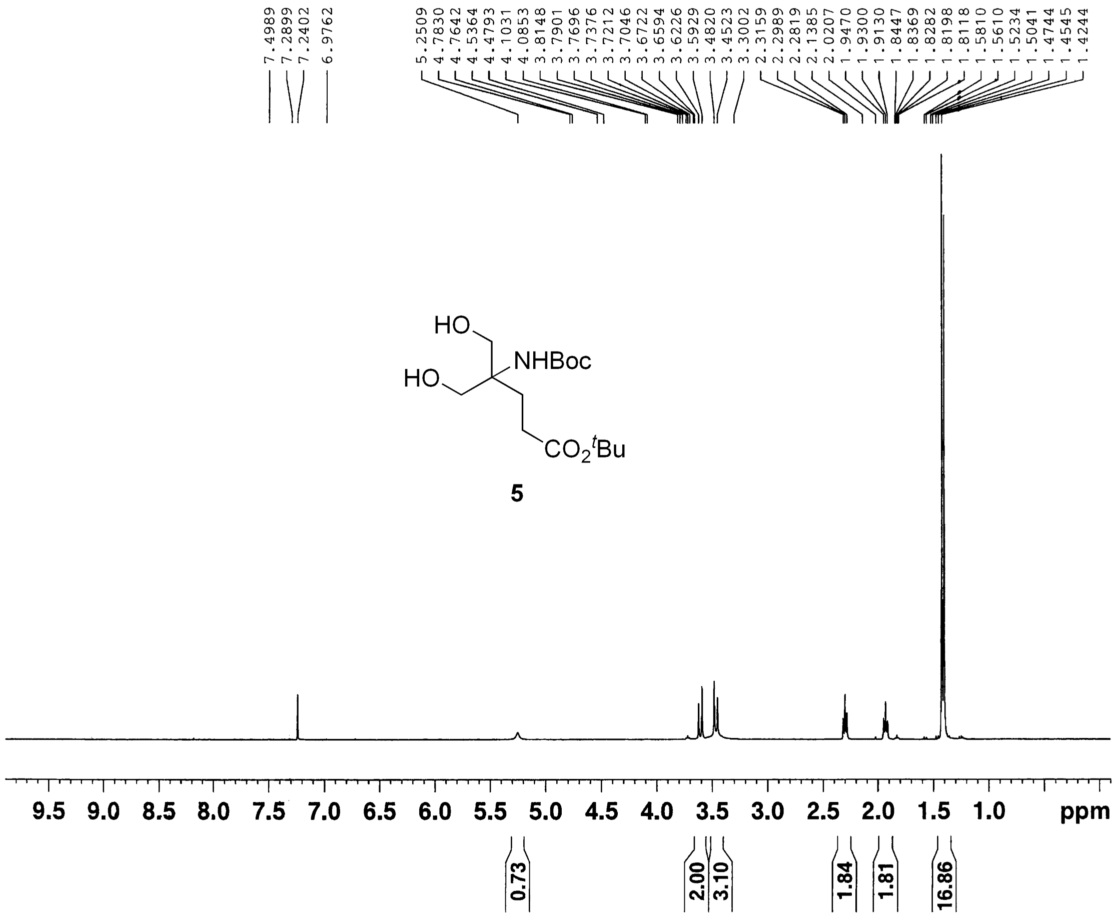

Current Data Parameters
NAME YH-I-166-1
EXPNO 1
PROCNO 1

F2 - Acquisition Parameters
Date_ 20141209
Time 13.15
INSTRUM dpx400
PROBHD 5 mm QNP 1H/13
PULPROG zg30
TD 65536
SOLVENT CDCl3
NS 16
DS 2
SWH 8278.146 Hz
FIDRES 0.126314 Hz
AQ 3.9584243 sec
RG 203.2
DW 60.400 usec
DE 6.00 usec
TE 300.0 K
D1 1.00000000 sec
MCREST 0.00000000 sec
MCWRK 0.01500000 sec

======== CHANNEL f1 ========
NUC1 1H
P1 12.60 usec
PL1 -6.00 dB
SFO1 400.1324710 MHz

F2 - Processing parameters
SI 32768
SF 400.1300174 MHz
WDW EM
SSB 0
LB 0.30 Hz
GB 0
PC 1.00

13C NMR (100 MHz, CDCl3)

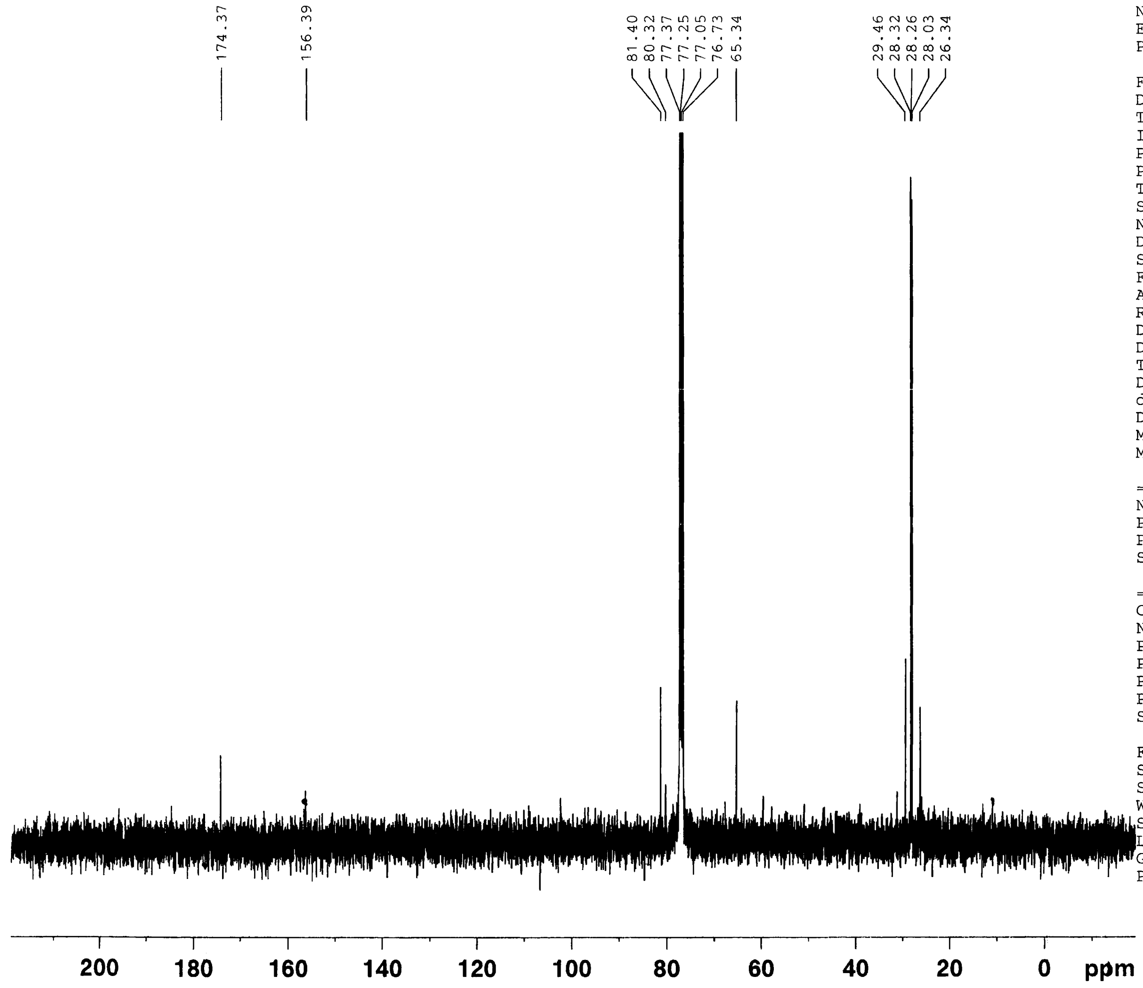

Current Data Parameters
NAME YH-I-166-1
EXPNO 6
PROCNO 1

F2 - Acquisition Parameters
Date_ 20150103
Time 14.05
INSTRUM dpx400
PROBHD 5 mm QNP 1H/13
PULPROG zgpg30
TD 65536
SOLVENT CDCl3
NS 2376
DS 4
SWH 23980.814 Hz
FIDRES 0.365918 Hz
AQ 1.3664756 sec
RG 9195.2
DW 20.850 usec
DE 6.00 usec
TE 300.0 K
D1 2.00000000 sec
d11 0.03000000 sec
DELTA 1.89999998 sec
MCREST 0.00000000 sec
MCWRK 0.01500000 sec

======== CHANNEL f1 ========
NUC1 13C
P1 8.00 usec
PL1 -5.00 dB
SFO1 100.6228298 MHz

======== CHANNEL f2 ========
CPDPRG2 waltz16
NUC2 1H
PCPD2 80.00 usec
PL2 -6.00 dB
PL12 10.00 dB
PL13 10.00 dB
SFO2 400.1316005 MHz

F2 - Processing parameters
SI 32768
SF 100.6127690 MHz
WDW EM
SSB 0
LB 1.00 Hz
GB 0
PC 1.40

1H NMR (400 MHz, CDCl3)

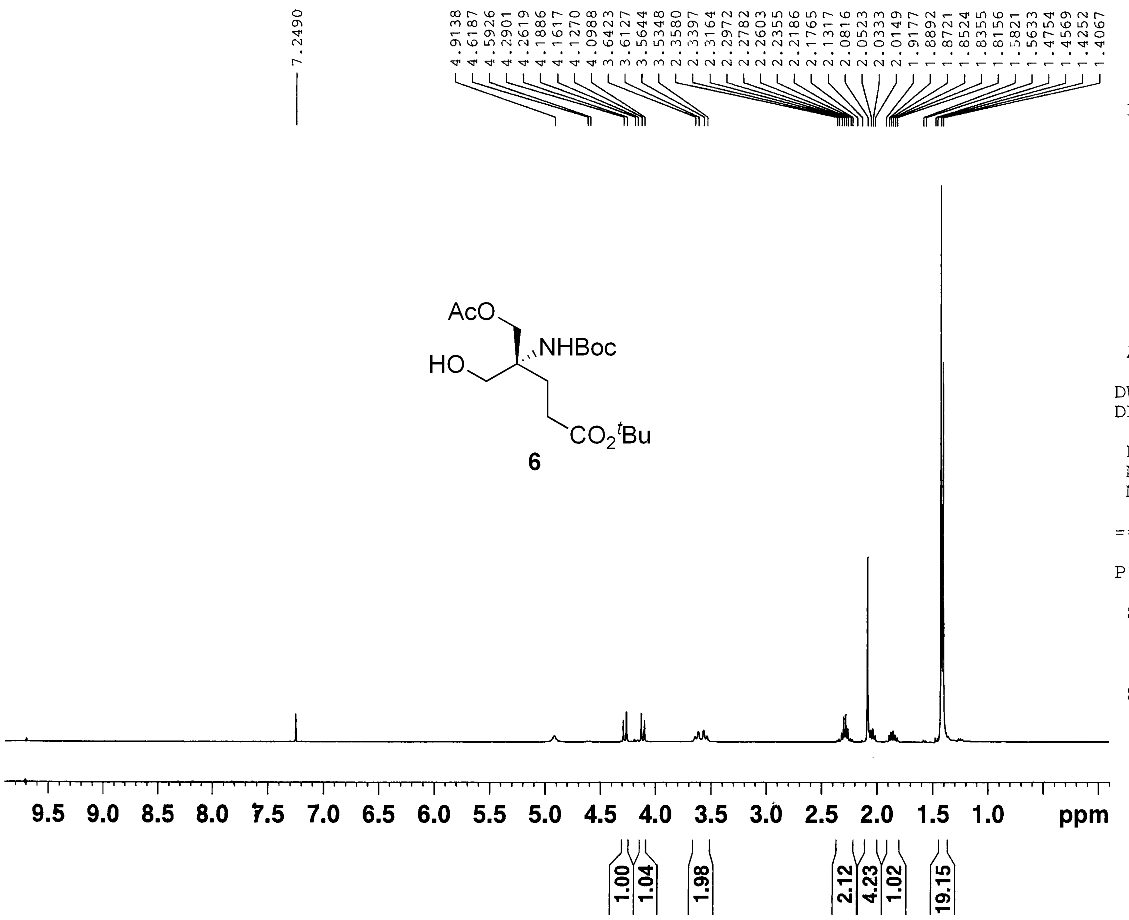

Current Data Parameters
NAME YH-I-178-1
EXPNO 1
PROCNO 1

F2 - Acquisition Parameters
Date_ 20141228
Time 13.05
INSTRUM dpx400
PROBHD 5 mm QNP 1H/13
PULPROG zg30
TD 65536
SOLVENT CDCl3
NS 16
DS 2
SWH 8278.146 Hz
FIDRES 0.126314 Hz
AQ 3.9584243 sec
RG 161.3
DW 60.400 usec
DE 6.00 usec
TE 300.0 K
D1 1.00000000 sec
MCREST 0.00000000 sec
MCWRK 0.01500000 sec

======== CHANNEL f1 ========
NUC1 1H
P1 12.60 usec
PL1 -6.00 dB
SFO1 400.1324710 MHz

F2 - Processing parameters
SI 32768
SF 400.1300139 MHz
WDW EM
SSB 0
LB 0.30 Hz
GB 0
PC 1.40

13C NMR (100 MHz, CDCl3)

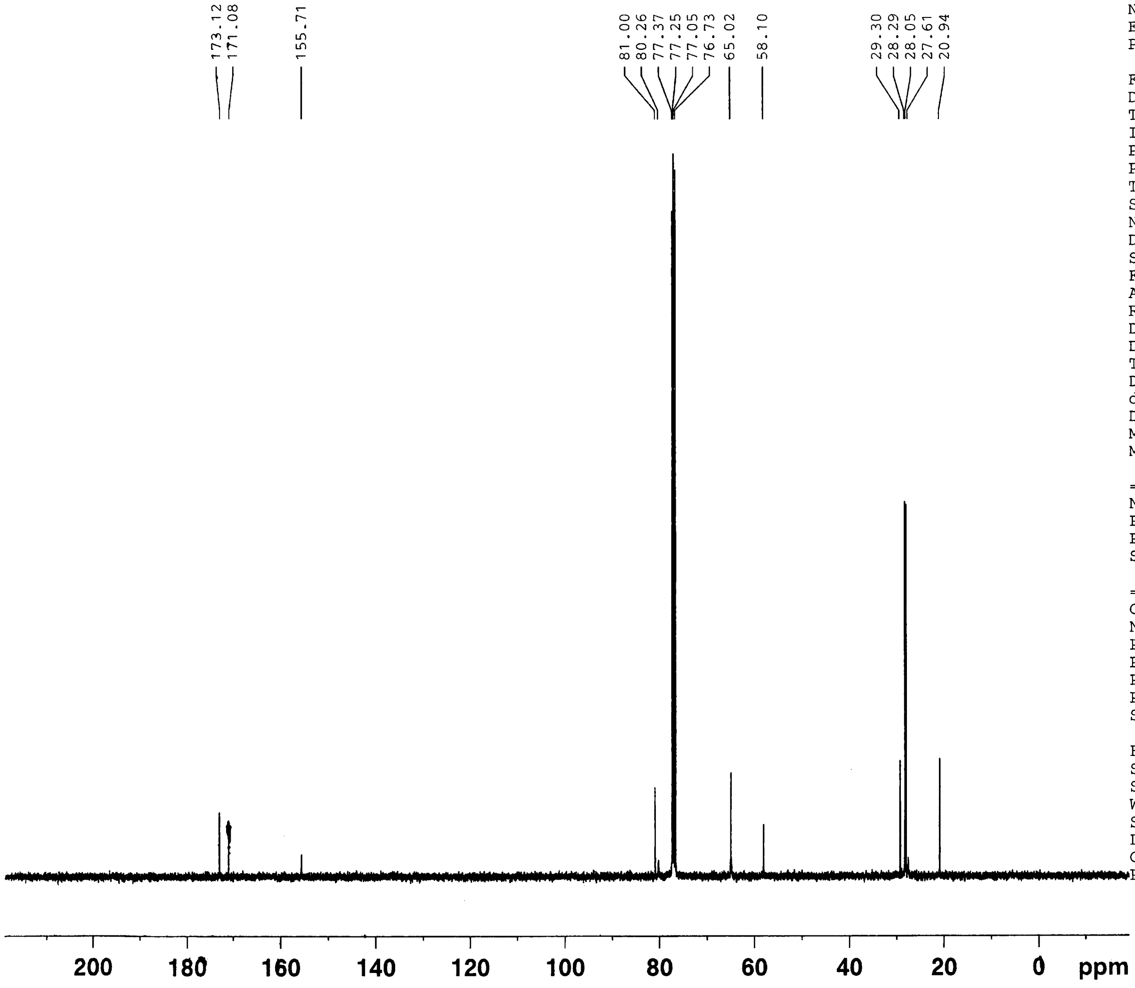

Current Data Parameters
NAME YH-I-178-1
EXPNO 2
PROCNO 1

F2 - Acquisition Parameters
Date_ 20141228
Time 14.12
INSTRUM dpx400
PROBHD 5 mm QNP 1H/13
PULPROG zgpg30
TD 65536
SOLVENT CDCl3
NS 1000
DS 4
SWH 23980.814 Hz
FIDRES 0.365918 Hz
AQ 1.3664756 sec
RG 18390.4
DW 20.850 usec
DE 6.00 usec
TE 300.0 K
D1 2.00000000 sec
d11 0.03000000 sec
DELTA 1.89999998 sec
MCREST 0.00000000 sec
MCWRK 0.01500000 sec

======== CHANNEL f1 ========
NUC1 13C
P1 8.00 usec
PL1 -5.00 dB
SFO1 100.6228298 MHz

======== CHANNEL f2 ========
CPDPRG2 waltz16
NUC2 1H
PCPD2 80.00 usec
PL2 -6.00 dB
PL12 10.00 dB
PL13 10.00 dB
SFO2 400.1316005 MHz

F2 - Processing parameters
SI 32768
SF 100.6127690 MHz
WDW EM
SSB 0
LB 1.00 Hz
GB 0
PC 1.00

1H NMR (400 MHz, CDCl3)

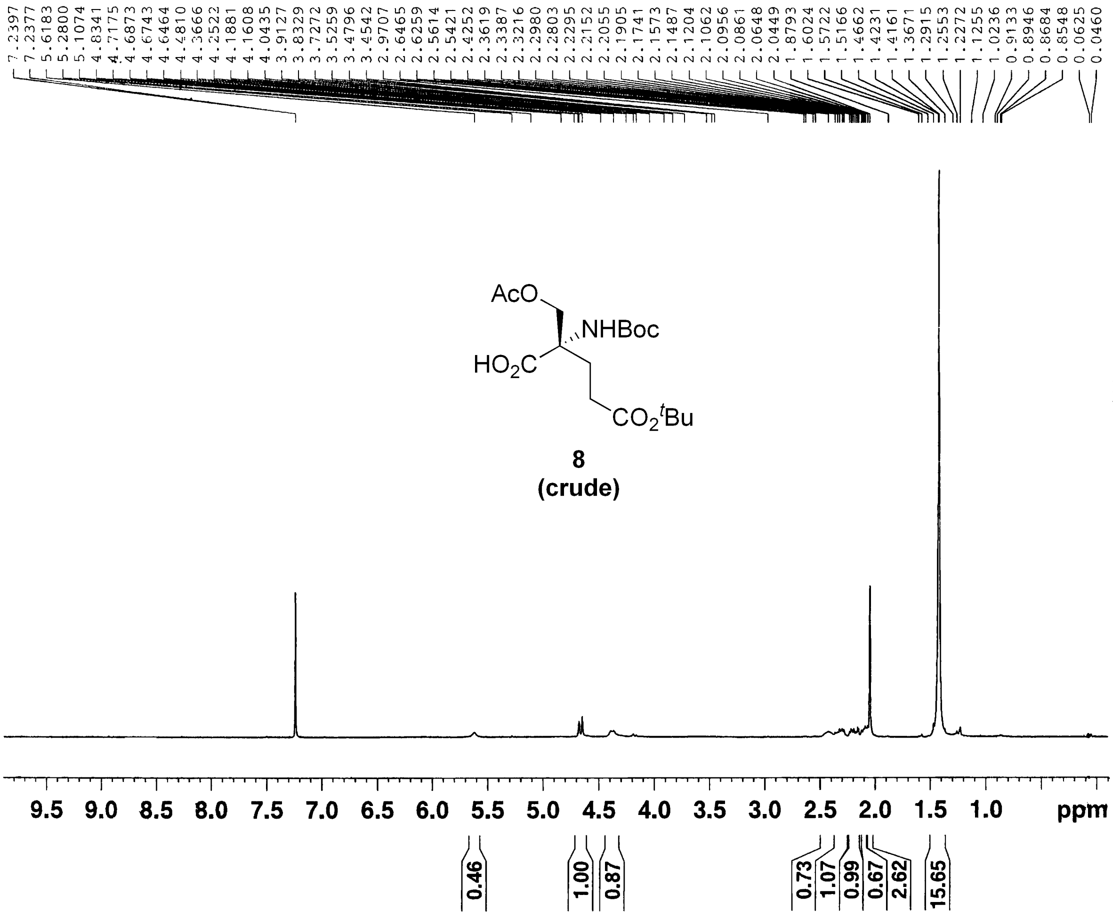

Current Data Parameters
NAME YH-I-178 crude
EXPNO 1
PROCNO 1

F2 - Acquisition Parameters
Date_ 20141222
Time 20.38
INSTRUM dpx400
PROBHD 5 mm QNP 1H/13
PULPROG zg30
TD 65536
SOLVENT CDCl3
NS 16
DS 2
SWH 8278.146 Hz
FIDRES 0.126314 Hz
AQ 3.9584243 sec
RG 574.7
DW 60.400 usec
DE 6.00 usec
TE 300.0 K
D1 1.00000000 sec
MCREST 0.00000000 sec
MCWRK 0.01500000 sec

======== CHANNEL f1 ========
NUC1 1H
P1 12.60 usec
PL1 -6.00 dB
SFO1 400.1324710 MHz

F2 - Processing parameters
SI 32768
SF 400.1300181 MHz
WDW EM
SSB 0
LB 0.30 Hz
GB 0
PC 1.00

1H NMR (400 MHz, D2O)

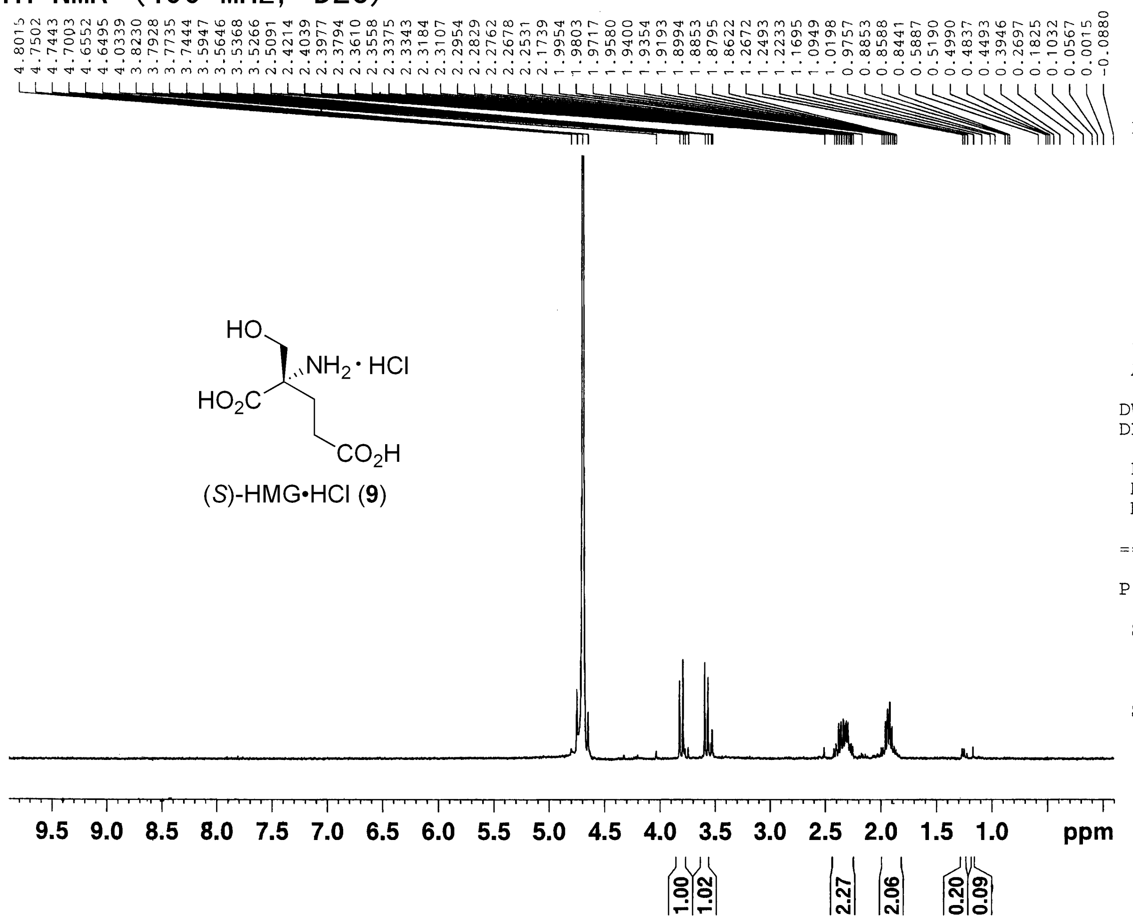

Current Data Parameters
NAME YH-I-178-2-1
EXPNO 1
PROCNO 1

F2 - Acquisition Parameters
Date_ 20141227
Time 10.50
INSTRUM dpx400
PROBHD 5 mm QNP 1H/13
PULPROG zg30
TD 65536
SOLVENT CDCl3
NS 16
DS 2
SWH 8278.146 Hz
FIDRES 0.126314 Hz
AQ 3.9584243 sec
RG 456.1
DW 60.400 usec
DE 6.00 usec
TE 300.0 K
D1 1.00000000 sec
MCREST 0.00000000 sec
MCWRK 0.01500000 sec

======== CHANNEL f1 ========
NUC1 1H
P1 12.60 usec
PL1 -6.00 dB
SFO1 400.1324710 MHz

F2 - Processing parameters
SI 32768
SF 400.1299995 MHz
WDW EM
SSB 0
LB 0.30 Hz
GB 0
PC 1.00

13C NMR (100 MHz, D2O)

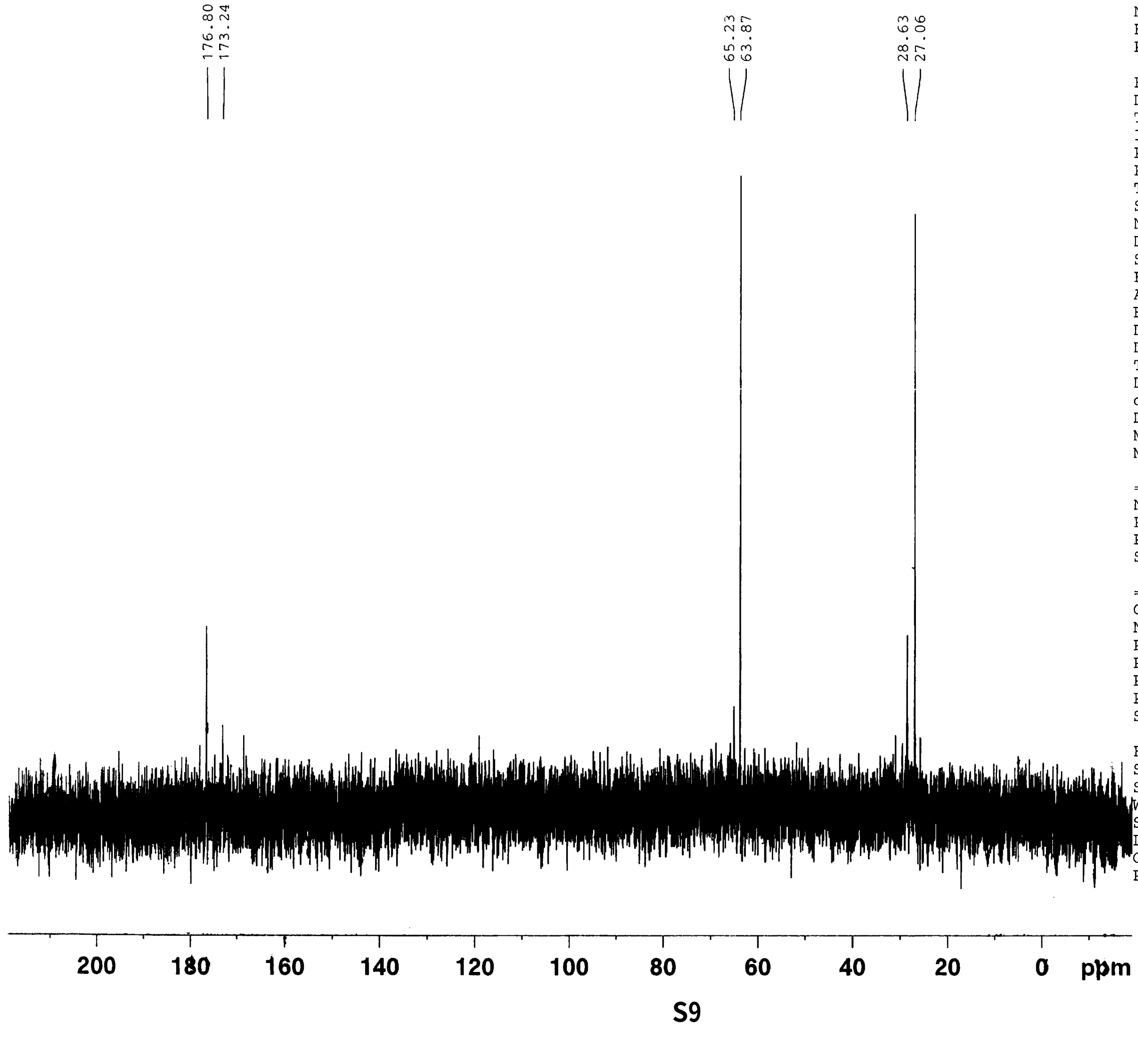

Current Data Parameters
NAME YH-I-178-2-1
EXPNO 2
PROCNO 1

F2 - Acquisition Parameters
Date_ 20141228
Time 6.37
INSTRUM dpx400
PROBHD 5 mm QNP 1H/13
PULPROG zgpg30
TD 65536
SOLVENT CDCl3
NS 10000
DS 4
SWH 23980.814 Hz
FIDRES 0.365918 Hz
AQ 1.3664756 sec
RG 4096
DW 20.850 usec
DE 6.00 usec
TE 300.0 K
D1 2.00000000 sec
d11 0.03000000 sec
DELTA 1.89999998 sec
MCREST 0.00000000 sec
MCWRK 0.01500000 sec

======== CHANNEL f1 ========
NUC1 13C
P1 8.00 usec
PL1 -5.00 dB
SFO1 100.6228298 MHz

======== CHANNEL f2 ========
CPDPRG2 waltz16
NUC2 1H
PCPD2 80.00 usec
PL2 -6.00 dB
PL12 10.00 dB
PL13 10.00 dB
SFO2 400.1316005 MHz

F2 - Processing parameters
SI 32768
SF 100.6127690 MHz
WDW EM
SSB 0
LB 1.00 Hz
GB 0
PC 1.40

1H NMR (400 MHz, CDCl3)

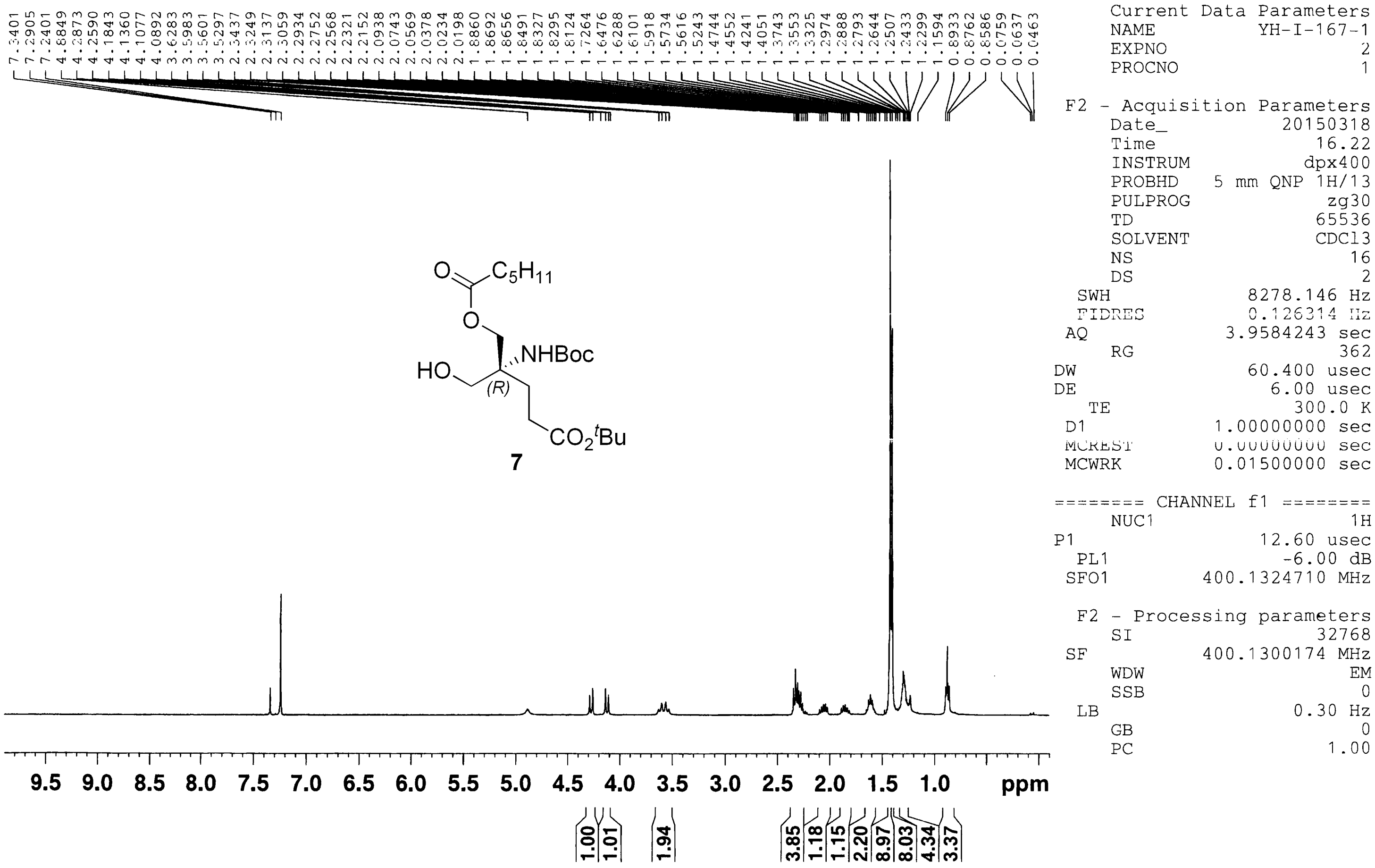

13C NMR (100 MHz, CDCl3)

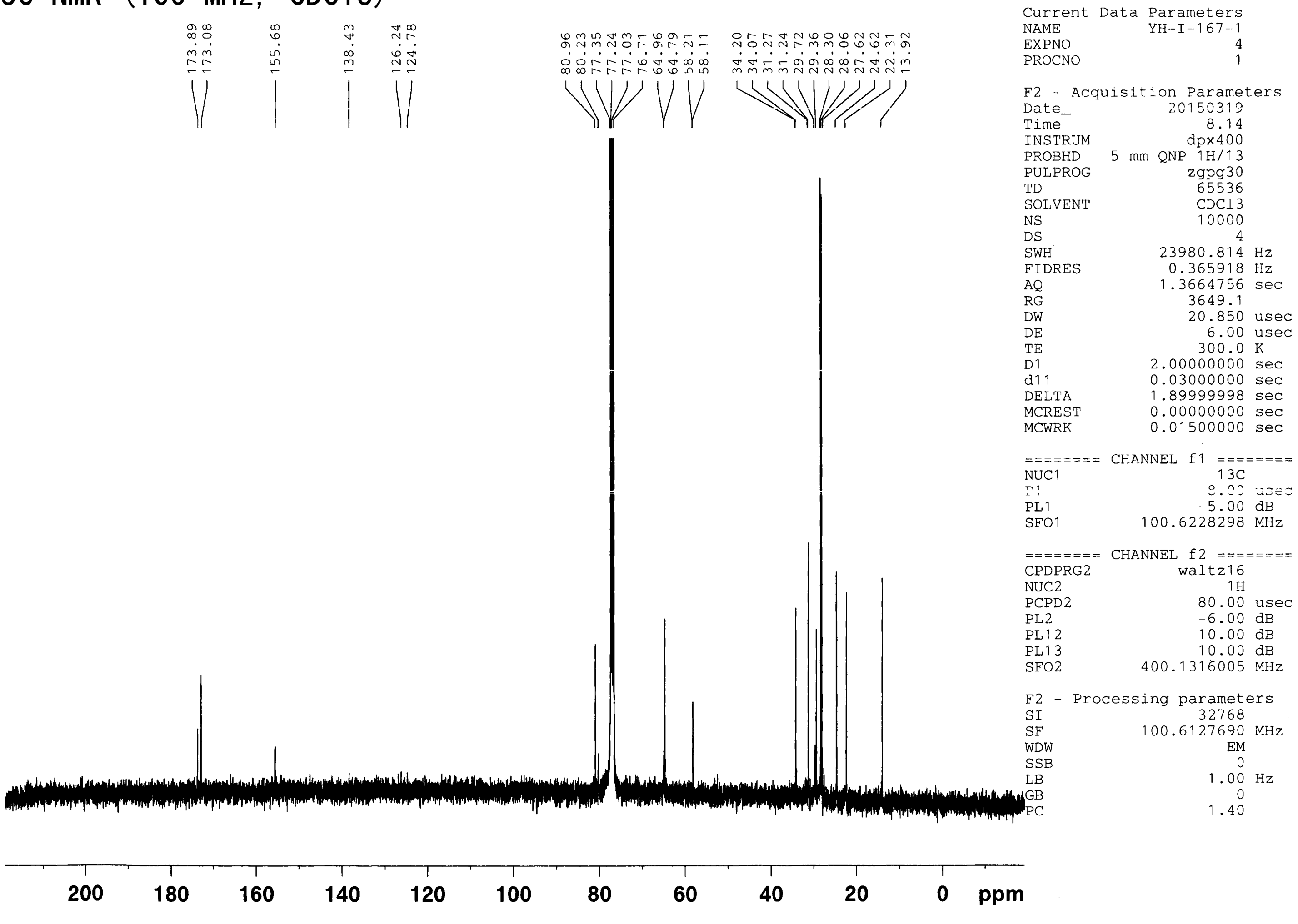

Chiral HPLC profile

Chiral HPLC analysis of monoacetate **6** for ee determination and resolution

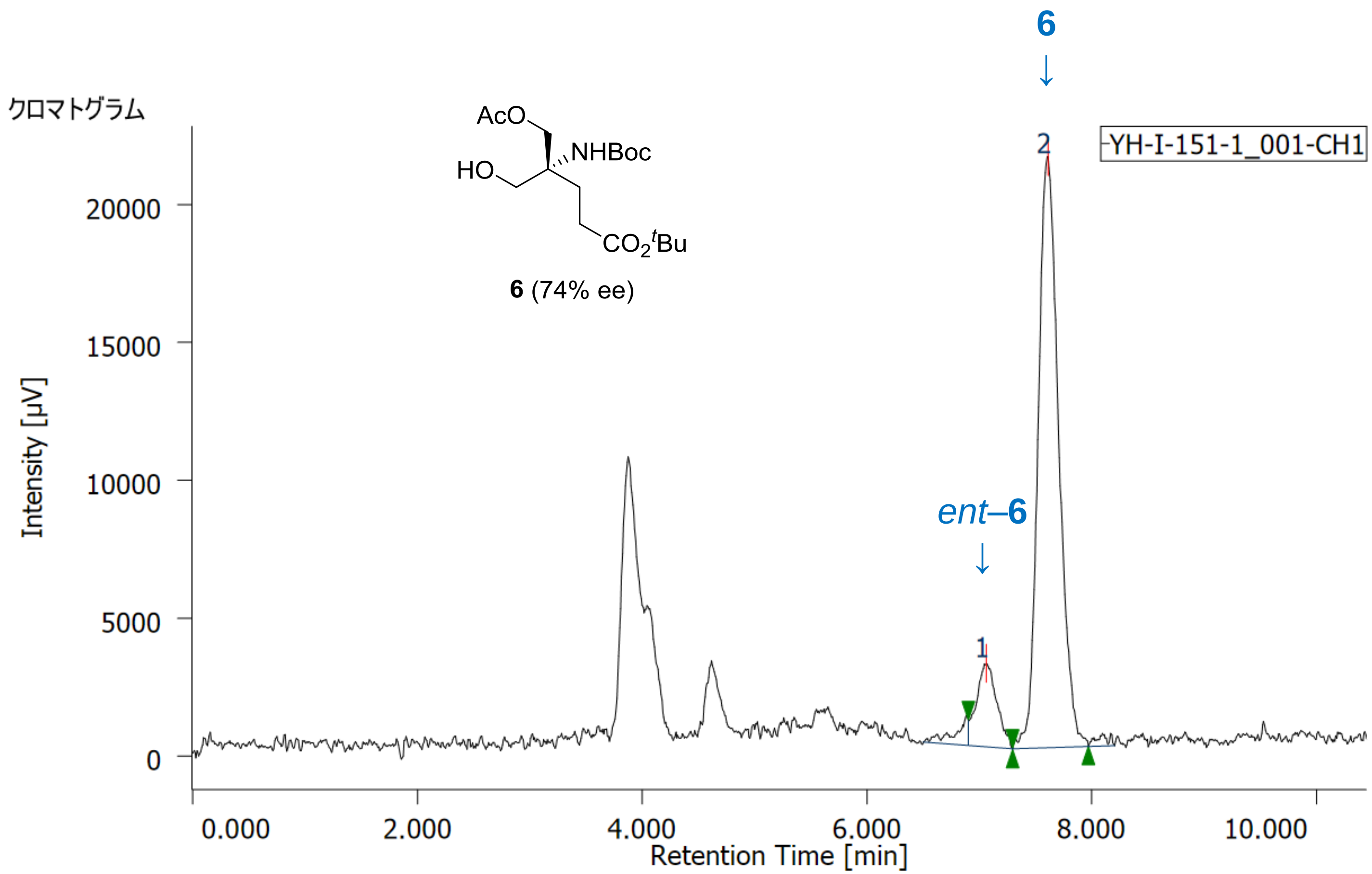

HPLC conditions:
CHIRALPAK IC column (0.46 × 25 cm), EtOH/hexane = 2:5, 1.0 mL/min, 40 ˚C, UV detection (210 nm)

Summary for previous synthetic study of HMG

Publication	Steps	Yield	Starting material
Zhang et al. 2001[1)]	3	34.6%	serine methyl ester
Aydillo et al. 2011[2)]	3	52.9%	N-Boc-serine methyl ester
Choudhury et al. 2002[3)]	9	20.7%	pyroglutamic acid
Kawasaki et al. 2003[4)]	6	21.2%	methyl 3-(chlorocarbonyl)propanoate
Tang et al. 2004[5)]	11	14.4%	methyl 5-bromolevulinate
Battistini et al. 2004[6)]	11	20.8%	N-Boc-3-pyrrolin-2-one
Hayes et al. 2006[7)]	5	35.9%	Garner's aldehyde
Miyaoka et al. 2006[8)]	8	46.1%	benzoic acid,<br>2-amino-2-hydroxymethylpropane-1,3-diol
Yiotakis et al. 2007[9)]	12	26.3%	valine
Martinkova et al. 2008[10)]	24	2.60%	1,2-di-O-isopropylidene-D-xylofuranose

References

1) Zhang J, Flippen-Anderson JL, Kozikowski AP (2001) A Tandem Michael Addition Ring-Closure Route to the Metabotropic Receptor Ligand α-(Hydroxymethyl)glutamic Acid and Its γ-Alkylated Derivatives. J Org Chem 66:7555-7559. doi:10.1021/jo010626n
2) Aydillo C, Jimenez-Oses G, Avenoza A, Busto JH, Peregrina JM, Zurbano MM (2011) A Domino Michael/Dieckmann Process as an Entry to α-(Hydroxymethyl)glutamic Acid. J Org Chem 76:6990-6996. doi:10.1021/jo201067n
3) Choudhury PK, Le Nguyen BK, Langlois N (2002) Stereoselective synthesis of (2S)-2-hydroxymethylglutamic acid, a potent agonist of metabotropic glutamate receptor mGluR3. Tetrahedron Lett 43:463-464. doi:10.1016/S0040-4039(01)02196-7
4) Kawasaki M, Namba K, Tsujishima H, Shinada T, Ohfune Y (2003) Efficient synthesis of optically active α-substituted glutamate analogs possessing α-hydroxymethyl and α-alkoxymethyl groups. Tetrahedron Lett 44:1235-1238. doi:10.1016/S0040-4039(02)02810-1
5) Tang G, Tian H, Ma D (2004) Asymmetric Strecker reaction of γ-keto acids. Facile entry to α-substituted and α,γ-disubstituted glutamic acids. Tetrahedron 60:10547-10552. doi:10.1016/j.tet.2004.07.100
6) Battistini L, Curti C, Zanardi F, Rassu G, Auzzas L, Casiraghi G (2004) Enantioselective Total Synthesis of (1R,3S,4R,5R)-1-Amino-4,5-

dihydroxycyclopentane-1,3-dicarboxylic Acid. A Full-Aldol Access to Carbaketose Derivatives. J Org Chem 69:2611-2613. doi:10.1021/jo035846a
7) Hayes CJ, Bradley DM, Thomson NM (2006) An efficient enantioselective synthesis of (2R)-hydroxymethyl glutamic acid and an approach to the (2R)-hydroxymethyl-substituted sphingofungins. J Org Chem 71:2661-2665. doi:10.1021/jo052408q
8) Miyaoka H, Yamanishi M, Hoshino A, Kinbara A (2006) (R)-4-Hydroxymethyl-2-phenyl-4,5-dihydrooxazol-4-ylmethyl acetate: chiral building block for the synthesis of optically active α-substituted α-amino acid derivatives. Tetrahedron 62:4103-4109. doi:10.1016/j.tet.2006.02.022
9) Yiotakis A, Magriotis PA, Vassiliou S (2007) A simple synthesis of the metabotropic receptor ligand (2S)-α-(hydroxymethyl)-glutamic acid and its Fmoc protected derivatives. Tetrahedron Asymmetry 18:873-877. doi:10.1016/j.tetasy.2007.03.028
10) Martinkova M, Gonda J, Raschmanova J, Uhrikova A (2008) Stereoselective synthesis of both enantiomers of α-(hydroxymethyl)glutamic acid. Tetrahedron Asymmetry 19:1879-1885. doi:10.1016/j.tetasy.2008.08.003
